# Antibiotic resistance in hospital-acquired ESKAPE-E infections in low- and lower-middle-income countries: a systematic review and meta-analysis

**DOI:** 10.1080/22221751.2022.2030196

**Published:** 2022-02-04

**Authors:** Olaniyi Ayobami, Simon Brinkwirth, Tim Eckmanns, Robby Markwart

**Affiliations:** a Unit for Healthcare Associated Infections, Surveillance of Antimicrobial Resistance and Consumption, Department of Infectious Disease Epidemiology, Robert Koch Institute, Berlin, Germany; b Jena University Hospital, Institute of General Practice and Family Medicine, Jena, Germany

**Keywords:** Antibiotic resistance, hospital-acquired infections, low-resource countries, Health equity

## Abstract

Antimicrobial resistance (AMR) and hospital-acquired infections (HAIs) are global health challenges. The burden of antibiotic resistance in HAIs is still unclear in low- and lower-middle-income countries (L-LMICs). This study summarizes recent data on antibiotic resistance in priority HAIs (ESKAPE-E) in L-LMICs and compares them with data from high-income countries (HICs). EMBASE, Web of Science, and Global Index Medicus were searched for studies on AMR patterns in HAIs published from 01/2010 to 10/2020. Random-effects meta-analyses were performed to obtain pooled estimates. In total, 163 eligible studies were included in the review and meta-analysis. The pooled methicillin resistance proportion in *Staphylococcus aureus* was 48.4% (95% confidence interval [95%CI] 41·7-55·2, n = 80). Pooled carbapenem resistance proportions were high in Gram-negative pathogens: *Escherichia coli*: 16·6% (95%CI 10·7-23·4, n = 60); *Klebsiella pneumoniae*: 34·9% (95%CI 24·6-45·9, n = 50); *Pseudomonas aeruginosa*: 37.1% (95%CI 24·6-45·9, n = 56); *Enterobacter* spp.: 51·2% (95%CI 27·5-74·7, n = 7); and *Acinetobacter baumannii (complex):* 72·4% (95%CI 62·1-81·7%, n = 36). A higher resistance proportions were observed for third-generation cephalosporins: *Klebsiella pneumoniae*: 78·7% (95%CI 71·5-85·2, n = 46); *Escherichia coli:* 78·5% (95%CI 72·1-84·2%, n = 58); and *Enterobacter* spp.: 83·5% (95%CI 71·9-92·8, n = 8). We observed a high between-study heterogeneity (I^2^  >  80%), which could not be explained by our set of moderators. Pooled resistance proportions for Gram-negative pathogens were higher in L-LMICs than regional and national estimates from HICs. Patients in resource-constrained regions are particularly affected by AMR. To combat the high resistance to critical antibiotics in L-LMICs, and bridge disparities in health, it is crucial to strengthen local surveillance and the health systems in general.

## Introduction

The declining effectiveness of antibiotics against bacterial infections is undermining the previous gains of the last century. Antimicrobial resistance (AMR) imposes significant health and economic toll on the individual and population health [1]. This challenge becomes more acute with diminishing numbers of new antibiotics in the drug development pipeline [2].

The leading cause of hospital-acquired infections (HAIs) worldwide includes *Enterococcus faecium*, *Staphylococcus aureus*, *Klebsiella pneumoniae*, *Acinetobacter baumannii*, *Pseudomonas aeruginosa*, *Enterobacter* spp., and *Escherichia coli* (ESKAPE-E) infections [3]. ESKAPE-E pathogens embody the top five bacterial families – with relevant intrinsic resistance and expansive capacity to acquire multi-drug resistance – prioritized as a global health threat urgently in need of new antibiotic research and development by the World Health Organization (WHO) [2]. Even though comprehensive data are scarce, previous studies suggest the low- and lower-middle-income (L-LMICs) are most likely to be the worst hit by the declining effectiveness of antibiotics due to pre-existing developmental challenges, including specific health system challenges [4,5].

A previous systematic review by Allengranzi and colleagues [5] showed that the burden of HAIs is higher in resource-limited countries than it is in the high-income countries (HICs). However, no study has comprehensively assessed the magnitude of antibiotic resistance pattern of HAIs in L-LMICs – much needed to benchmark, plan and evaluate interventions. Therefore, this systematic review estimated the proportion of antibiotic resistance in hospital-acquired ESKAPE-E infections in L-LMICs as well as the associated mortality. We further compared these estimates from L-LMICs with national and regional estimates from high-income countries.

## Methods

This systematic review and meta-analysis were conducted according to a protocol published a priori in the Prospective Register for Systematic Reviews (CRD42020210481) and followed the updated guidelines from the Preferred Reporting Items for Systematic Reviews and Meta-analyses (PRISMA) statement [6].

### Study outcomes

The primary outcome of this study is the antibiotic resistance proportion in bacterial isolates from patients with hospital-acquired infections in L-LMICs (World Bank classification) [7]. The antibiotic resistance proportion is defined as the total number of isolates tested as resistant or intermediate against a given antibiotic among all tested isolates. We analysed antibiotic resistance proportions in ESKAPE-E organisms: vancomycin-resistant *E. faecium* (VRE), methicillin-resistant *S. aureus* (MRSA), vancomycin-resistant *S. aureus* (VRSA), vancomycin-resistant MRSA (VR-MRSA), carbapenem-resistant *K. pneumoniae* (CRKP), third-generation cephalosporin-resistant *K. pneumoniae* (TGCR-KP), carbapenem-resistant *A. baumannii* (complex) (CRAB), carbapenem-resistant *P. aeruginosa* (CRPA), carbapenem-resistant *Enterobacter* spp. (CREnt), third-generation cephalosporin-resistant *Enterobacter* spp. (TGCR-Ent), carbapenem-resistant *E. coli* (CREC) and third-generation cephalosporin-resistant *E. coli* (TGCR-EC). The secondary outcome is the attributable or all-cause mortality of patients with HAIs due to any of the above-mentioned antibiotic-resistant bacteria.

### Search strategy

The electronic databases EMBASE (including MEDLINE databank), Web of Science, and Global Index Medicus (Africa, Eastern Mediterranean, South-East Asia, Latin America and the Caribbean, and Western Pacific Region) were searched for studies published from 1st January 2010 to 22nd October 2020 (date of the last search). The detailed search strategy is provided in the Supplementary document. Title, abstract and full-text screening were independently performed by at least two authors (OA, SB, RM) using *Covidence*, a software tool recommended by the Cochrane Community [8]. All disagreements were resolved by discussion or by a third reviewer.

### Study selection criteria

Studies were included if they met the following criteria: (i) studies reported quantitative data (number of resistant isolates + number of tested isolates) for at least one of the predefined antibiotic-pathogen combinations with at least 10 tested isolates per combination; (ii) bacterial isolates were derived from hospital-acquired infections; (iii) the study was conducted in low- or lower-middle-income countries; (iv) data collection was completed after 12/2009; (v) the patient cohort represented a largely unselected population in the healthcare facility, i.e. not only high-risk patients (such as low birth weight neonates) or those with a specific underlying disease; (vi) the study was written in English, French, German, Spanish or Portuguese. Studies with the following design were excluded: narrative or systematic reviews, case reports, and case series.

### Data extraction and risk of bias assessment

The data of all eligible studies were independently extracted by at least two authors (OA, SB, RM) using standardized forms. All disagreements were resolved through discussion or by a third reviewer. The data extraction included the primary and secondary outcomes and the following study characteristics: First author, year of publication, study period, country, city, WHO region, continent, national income level category, study design, regional or national representativeness, hospital setting, age group, hospital type, antimicrobial susceptibility testing (AST) guideline used, and HAI type. In cases where studies reported resistance data for several antibiotics from one antibiotic class (e.g. meropenem, imipenem, and ertapenem for carbapenem resistance), the data with the highest resistance proportion were extracted. All included studies were assessed for risk of bias by at least two authors (OA, SB, RM). The risk of bias was judged for three domains: (i) national or regional representativeness of included patients/HAI isolates, (ii) sample selection method, and (iii) use of a sound microbiological method for pathogen identification and AST. The risk of bias for national or regional representativeness was judged as “low” if the study explicitly used an appropriate method. The risk of bias for sample selection was judged as “low” if the study included all patients/HAI isolates in the study period (e.g. by consecutive inclusion of all patients/isolates) or used some form of random selection. If the study used established methods for pathogen identification and AST (e.g. automated systems, such as Vitek, Phoenix, or BacTec, as well as AST guidelines, such as CLSI and EUCAST), the risk of bias was judged as “low”. Otherwise, the risk of bias was adjudged as high for the three criteria.

### Statistical analysis

For data analysis and presentation, data were grouped according to the antibiotic-pathogen combination (e.g. carbapenem resistance in *E. coli* isolates). Pooled estimates were calculated using random-effects models with a Tukey Double Arcsine transformation [9] of the raw proportions. The DerSimonian-Laird estimator was used to define τ² (between-study variance). *I*^2^ statistics quantified the statistical heterogeneity of the selected studies. We performed pre-planned subgroup analyses for WHO regions, national income level, hospital setting, and age groups (adults vs. paediatrics i.e. age less than 18years). Subgroups were only included in subgroup analyses if they included at least three studies. In order to study the potential influence of covariates (moderators) on the pooled effect sizes and the between-study variance, we conducted a moderator analysis [10]. The following moderators were considered: Study design (multicentre, single centre), WHO region, national income level, patient age group and HAI type. All statistical analyses were performed using “*R*” (v. 4.0.2) [11] using the packages *meta* [12] (v. 4.18-0) and *metafor* [13] (v. 2.4-0).

## Results

**Figure 1.**
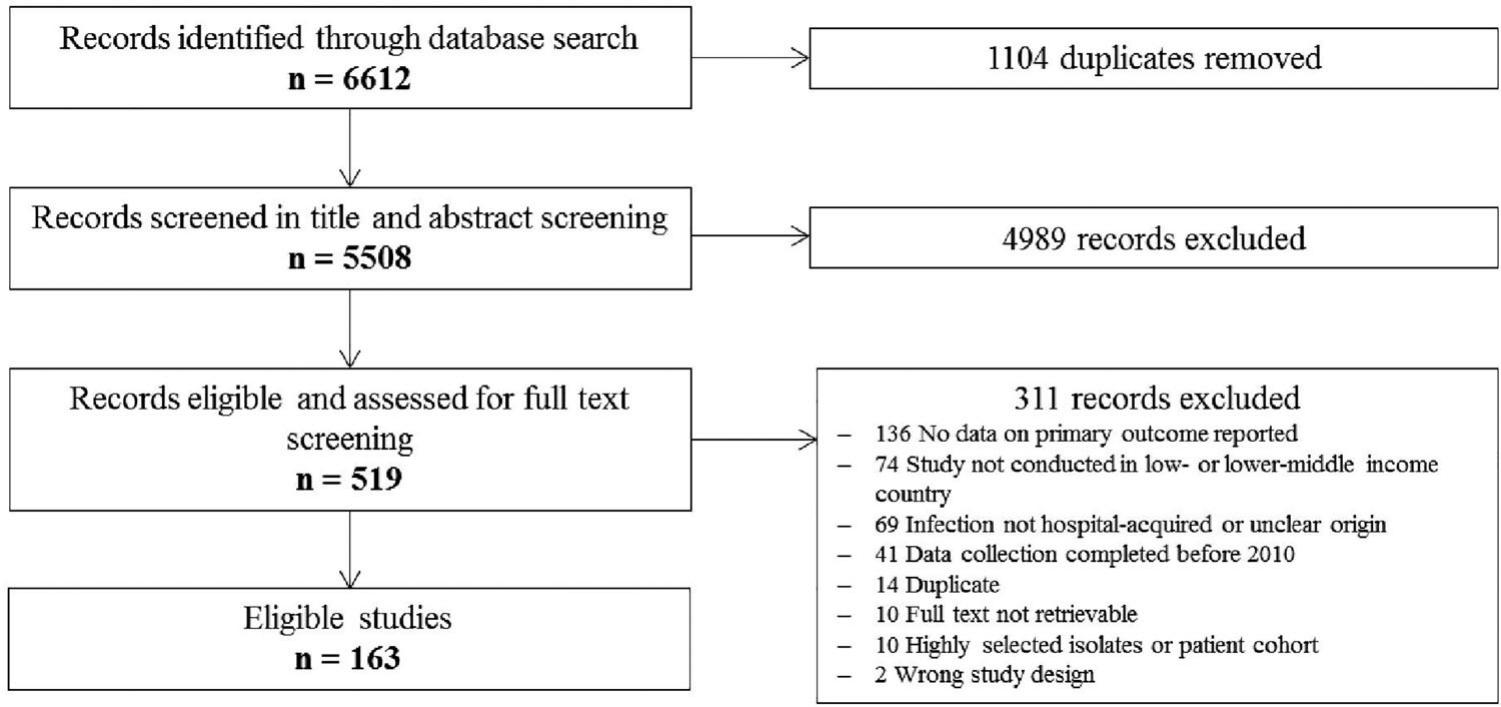
Flow chart of study selection.

**Figure 2.**
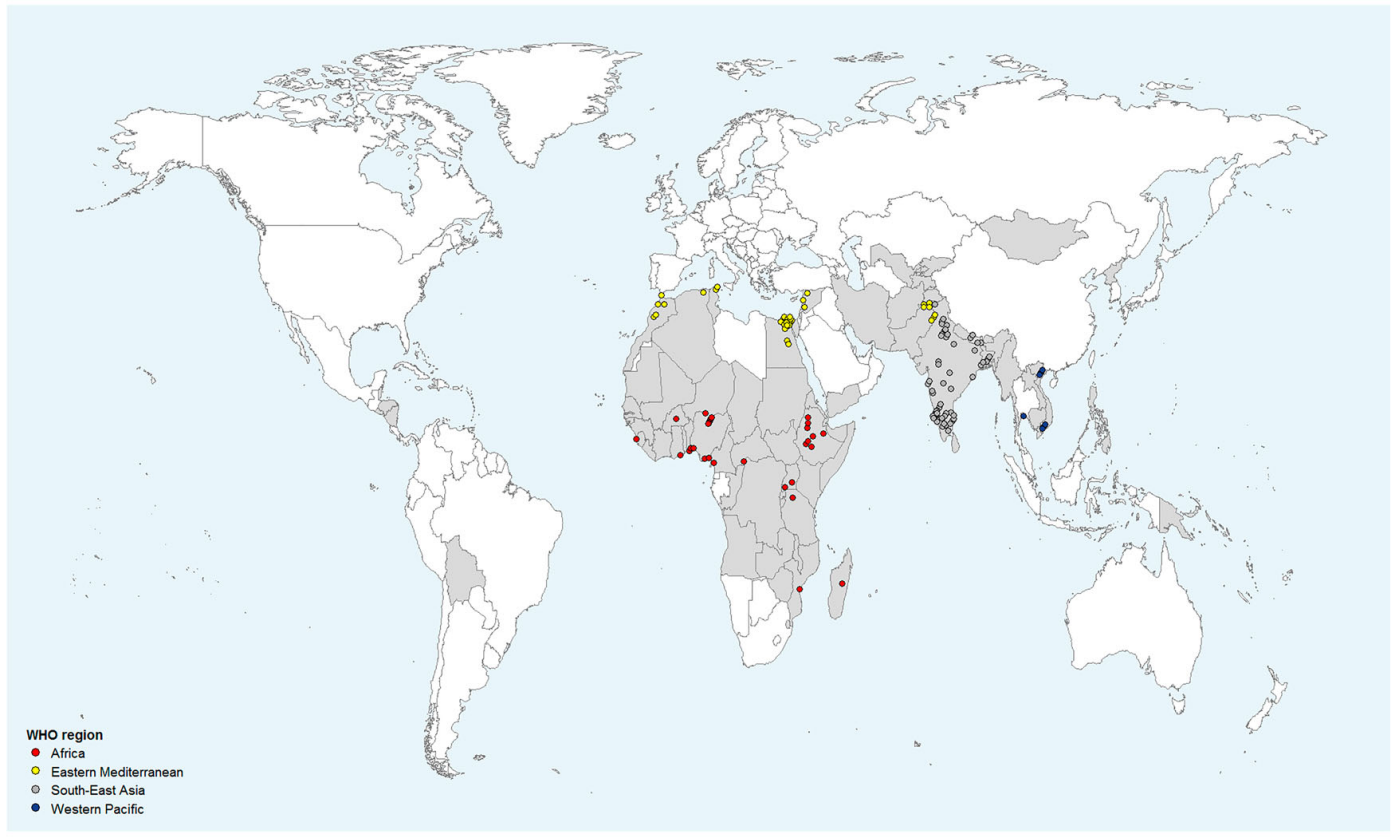
Geographical distribution of included studies by country.

In total, 163 studies were included in this systematic review and meta-analysis (see Figure 1 and Supplementary Table 1). Comprehensive overviews of the results of this study are presented in Tables 1 and 2, and forest plots of the main analyses in Supplementary Figures 1–11. A comparison of our data with regional and national resistance data from upper-middle and HICs is presented in Table 3. The main characteristics of the included studies are summarized in Supplementary Table 1. The 163 included studies were conducted in 24 different countries (eight in Low-income and 16 in Lower-middle-income countries) and were distributed over four WHO regions: African Region (31), Eastern Mediterranean Region (56), South-East Asian Region (67), and Western Pacific Region (9) (Figure 2). In our study set, the most represented countries were India (*n* = 61) and Egypt (*n* = 36). The majority of studies were conducted hospital-wide (*n* = 78) or in intensive care units (ICUs: *n* = 64), and the remaining 21 studies were conducted in specialized wards with or without ICUs.

**Table 1.** Summary of resistance proportions in HAIs reported from studies conducted in low- and lower-middle-income countries.

Pathogen	Pooled proportion in % (95% CI)	Number of studies, range of study estimates (median %)	Heterogeneity (%)
***Gram-positive pathogens***			
Methicillin resistance in ***S. aureus (MRSA)***	**48.4** (41.7–55.2)	*n* = 80 0–100 (47.9)	*I*^2^ = 97.3, *Q* = 2935, *p* < 0.0001
Vancomycin resistance in ***S. aureus***	**0.6%** (0–1.8)	*n* = 39 0–30.4% (0%)	*I*^2^ = 82.5%, *Q* = 217, *p* < 0.01
Vancomycin resistance in ***MRSA***	**1.7%** (0–9.5)	*n* = 23 0–58.8% (0%)	*I*^2^ = 98.4%, *Q* = 1338, *p* < 0.01
Vancomycin resistance in ***E. faecium***	**–**	*n* = 1 23%	–
***Carbapenem resistance proportions in Gram-negative pathogens***			
***K. pneumoniae***	**34.9** (24.6–45.9)	*n* = 50 0–100 (33.3)	*I*^2^ = 95.3, *Q* = 1043, *p* < 0.01
***A. baumannii (complex)***	**72.4** (62.1–81.7)	*n* = 36 0–100 (83.4%)	*I*^2^ = 96.3, *Q* = 943, *p* < 0.01
***P. aeruginosa***	**37.1** (29.3–45.2)	*n* = 56 0–88.5 (41.2)	*I*^2^ = 94.9, *Q* = 1071, *p* < 0.01
***E. coli***	**16.6** (10.7–23.4)	*n* = 60 0–100 (14.0)	*I*^2^ = 95.9, *Q* = 1431, *p* < 0.01
***Enterobacter* spp.**	**51.2** (27.5–74.7)	*n* = 7 5.4–97.4 (51.1)	*I*^2^ = 96.2, *Q* = 156, *p* < 0.01
***Third-generation cephalosporin resistance proportions in Gram-negative pathogens***			
***K. pneumoniae***	**78.7** (71.5–85.2)	*n* = 46 16.7–100 (75.0%)	*I*^2^ = 91.2, *Q* = 512, *p* < 0.01
***E. coli***	**78.5** (72.1–84.2)	*n* = 58 19.7–100 (79.5%)	*I*^2^ = 94.6, *Q* = 1053, *p* < 0.01
***Enterobacter* spp.**	**83.5** (71.9–92.8)	*n* = 8 67.8–100 (79.8)	*I*^2^ = 85.3, *Q* = 48, *p* < 0.01

CI, confidence interval.

**Table 2.** Pooled resistance proportions (% with 95% confidence interval and number of studies) in hospital-acquired infections in low- and lower-middle-income countries per WHO region.

Pathogen	WHO Africa	WHO Eastern Mediterranean	WHO South-East Asia	WHO West-Pacific
***S. aureus***				
Methicillin resistance in ***S. aureus (MRSA)***	**49.6** (37.4–61.7, *n* = 22)	**56.6** (43.2–69.5, *n* = 21)	**41.0** (29.9–52.5, *n* = 33)	**63.2** (42.8–81.6, *n* = 4)
Vancomycin resistance in ***S. aureus***	**0.6%** (0–3.7, *n* = 6)	**3.9%** (0–14.8, *n* = 10)	**0.0%** (0–0.7, *n* = 23)	–
Vancomycin resistance in ***MRSA***	**0.6%** (0–5.6, *n* = 3)	**3.1%** (0–10.5, *n* = 7)	**1.5%** (0–16.4, *n* = 13)	–
***Carbapenem resistance proportions in Gram-negative pathogens***				
***K. pneumoniae***	**28.4** (0–87.0, *n* = 4)	**54.1** (32.5–74.9, *n* = 13)	**29.6** (18.4–42.0, *n* = 27)	**26.3** (1.7–63.8, *n* = 6)
***A. baumannii (complex)***	**1·6** (0–10.8, *n* = 3)	**77.0** (53.8–94.2, *n* = 12)	**76.8** (63.1–88.3, *n* = 13)	**82.8** (71.5–91.8, *n* = 8)
***P. aeruginosa***	**13**.**7** (0–57.3, *n* = 5)	**32**.**7** (19.6–47.1, *n* = 17)	**38**.**4** (28.3–49.0, *n* = 27)	**60**.**6** (43.9–76.2, *n* = 7)
***E. coli***	**7**.**1** (0–19.4, *n* = 11)	**16**.**5** (6.4–29.5, *n* = 14)	**20**.**7** (12.3–30.4, *n* = 33)	**12**.**1** (0–71.7, *n* = 2)
***Enterobacter* spp.**	**5**.**3** (0–21.2, *n* = 1)	**65**.**3** (21.0–98.0, *n* = 2)	**57**.**8** (16.6–93.4, *n* = 3)	**54**.**6** (24.2–83.3, *n* = 1)
***Third-generation cephalosporin resistance proportions in Gram-negative pathogens***				
***K. pneumoniae***	**61**.**8** (45.9–76.5, *n* = 9)	**93**.**5** (85.6–98.7, *n* = 8)	**79**.**7** (70.3–87.8, *n* = 26)	**70**.**2** (33.8–96.3, *n* = 3)
***E. coli***	**58**.**3** (45.5–70.6, *n* = 16)	**90**.**9** (72.9–100, *n* = 8)	**83**.**0** (76.5–88.8, *n* = 32)	**91**.**4** (62.2–100, *n* = 2)
***Enterobacter* spp.**	**80**.**1** (65.7–91.6, *n* = 2)	**93**.**8** (75.1–100, *n* = 1)	**84**.**1** (66.4–96.2, *n* = 4)	**72**.**7** (42.1–95.6, *n* = 1)

**Table 3.** Comparison of resistance proportions in ESKAPE-E organisms between resource-limited countries and upper-middle-income and high-income countries^a^.

Pathogen	L-LMIC countries (pooled estimates)	United States^b^ [41]	ReLAVRA^c^ [50]	EU/EEA^d^ [39]	Germany^e^	Japan^f^ [42]	China^g^ [43,51]
***S. aureus***							
**MRSA**	48.2%	40.6%	47.7%	15.5%	9.9%	46.1%	31.4%
**VRSA**	0.6%	–	–	–	0.0%	0.0%	0.0%
**VR-MRSA**	1.7%	0.1%	–	–	–	0.0%	–
***Carbapenem resistance in Gram-negative pathogens***							
***K. pneumoniae***	34.8%	4.7%	16.5%	7.9%	0.6%	0.5%	20.9%
***P. aeruginosa***	37.1%	13.3%	–	16.5%	12.9%	20.0%	23.6%
***E. coli***	16.6%	0.6%	–	0.3%	0.0%	0.2%	1.9%
***Enterobacter* spp.**	51.2%	4.6%	–	–	0.5%	4.7%	–
***A. baumannii***	72.4%	33.9%	–	32.6%	4.7%	1.8%	70.7%
***Third-generation cephalosporin resistance in Gram-negative pathogens***							
***K. pneumoniae***	78.7%	22.9%	62.2%	31.3%	13.1%%	11.4%	47.3%
***E. coli***	78.6%	22.0%	–	15.1%	11.8%	28.9%	59.3%
***Enterobacter* spp.**	83.5%	9.5%	–	–	25.6%	37.2%	–

L-LMICs, Low- and lower-middle-income countries.

^a^ All data are for nosocomial infections unless otherwise stated.

^b^ National surveillance data (2019), *Acinetobacter* and *Klebsiella* not speciated.

^c^ Regional surveillance including surveillance data from 19 South American countries (2016), *K. pneumoniae* data included both nosocomial and non-nosocomial isolates.

^d^ Regional surveillance data (2019) from the *European Antimicrobial Resistance Surveillance Network* (EARS-Net), invasive nosocomial and non-nosocomial infections (CSF + bloodstream), *Acinetobacter* not speciated.

^e^ National surveillance data (2019) from the *Antibiotika-Resistenz-Surveillance* (ARS).

^f^ National surveillance data (2019), *Acinetobacter* not speciated.

^g^ National surveillance data for 2017 except MRSA which included only 2019 data, nosocomial and non-nosocomial infections, *Acinetobacter* not speciated.

The overall risk of bias was judged as “moderate” (88/163, 54.0%) or “high” (74/163, 45.4%) in almost all studies (162/183, 99.4%), while it was judged as “low” in only one study (Supplementary Table 2). Apart from two studies from Benin [14] and Ghana [15] that explicitly included nationally representative hospital/patient samples, the risk of bias for the regional or national representativeness of the studied hospital population was assessed as “high” in all studies (*n* = 161).

**Figure 3.**
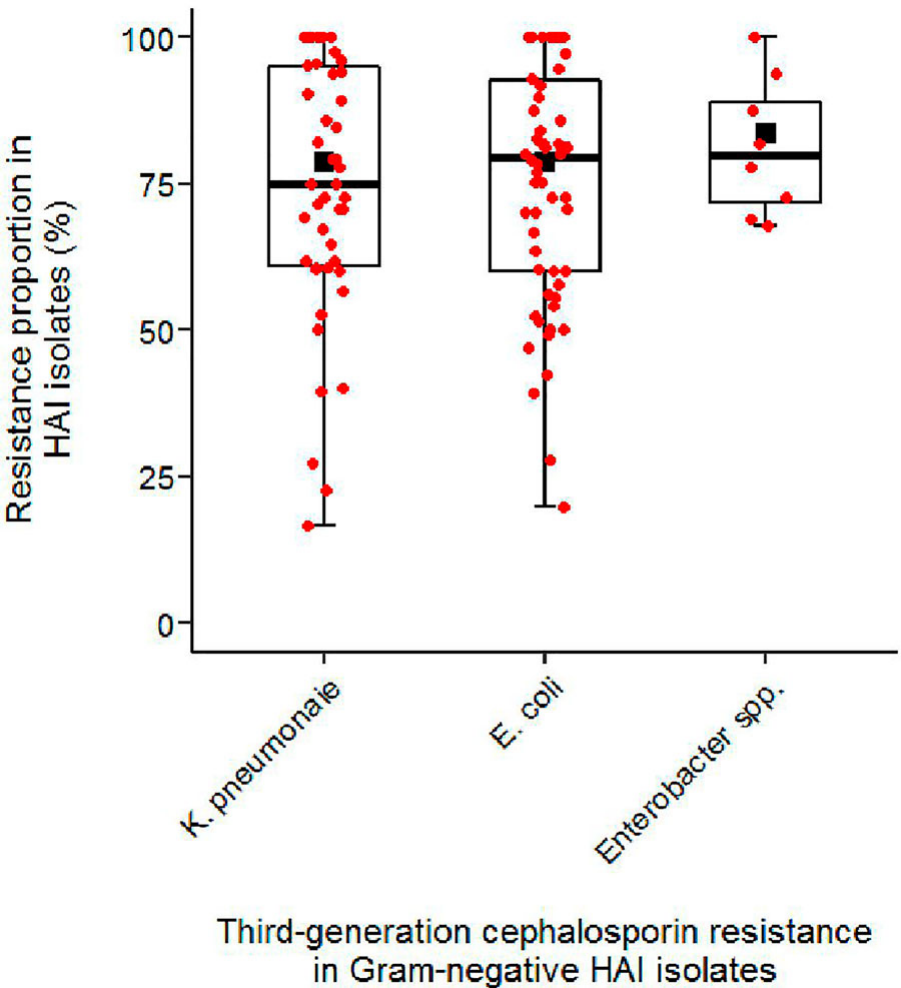
Third-generation resistance proportions in Gram-negative pathogens from hospital-acquired infections in low- and lower-middle-income countries (2010–2020). Box plots indicate individual study estimates of third-generation cephalosporin resistance proportions (red dots) and range for first and third quartile. Medians are indicated as a black line and pooled estimates from meta-analysis are displayed as black squares. Whiskers indicate lower and upper end of distribution. Resistance proportions are expressed as percentages (%) of third-generation cephalosporin-resistant or -non-susceptible isolates among all tested isolates.

The pooled TGCR proportion was 78.5% (95%CI 72.1–84.2%, range: 19.7–100%, median: 79.5%, *n* = 58), 78.7% (95%CI 71.4–85·2%, range: 16.7–100%, median:75.0%, *n* = 46) and 83.5% (95%CI 71.9–92.8%, range: 67.9–100%, *n* = 8) for *E. coli*, *K. pneumoniae* and *Enterobacter* spp.*,* respectively (Table 1, Figure 3).

Regional subgroup analyses revealed that pooled TGCR proportions in *E. coli* HAI isolates were statistically significantly lower in WHO Africa (58.3% [95%CI 45.5–70.6%], *n* = 18) compared to WHO Eastern Mediterranean (90.9% [95%CI 72.9–100%], *n* = 8; *Q* = 8. 02, *p* = 0.005) and WHO South-East Asia (83.0% [95%CI 76.5–88.8%], *n*  = 16; *Q* = 12.73, *p* = 0.0004). Similarly, pooled TGCR proportions were also lower in *K. pneumoniae* in WHO Africa (61.8% [95%CI 45.9–76.5%], *n* = 9) compared to WHO Eastern Mediterranean (93.5% [95%CI 85.6–98.7%], *n* = 8; *Q* = 14.85, *p* = 0.0001) and WHO South-East Asia (79.7% [95%CI 70.3–87.8%], *n* = 26; *Q* = 4.05, *p* = 0.0443). While no significant differences in pooled TGCR proportion between adults and paediatrics (< 18 years) were found for *E. coli* (*Q* = 0.95, *p* = 0.3292), TGCR proportion in *K. pneumoniae* were higher in paediatrics (88.4% [95%CI 76.2–96.9%], *n* = 6) compared to adults (67.6% [95%CI 49.9–83.2%], *n*  = 11; *Q* = 4.56, *p* = 0.0327).

**Figure 4.**
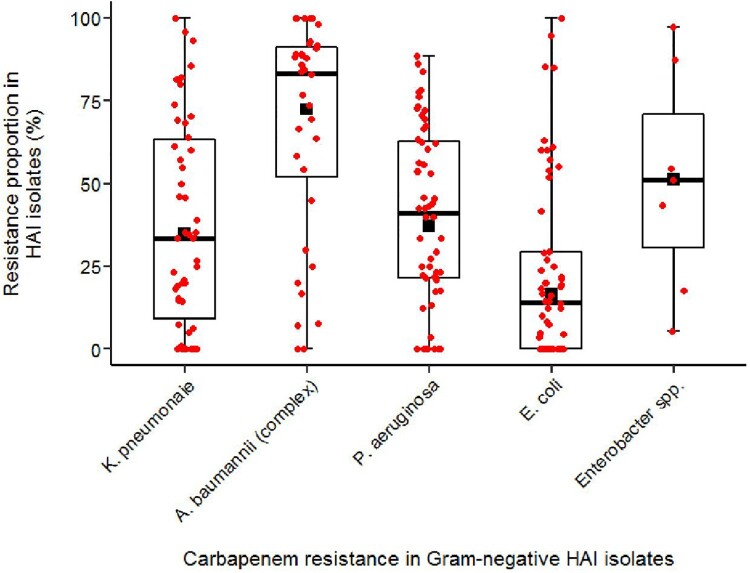
Carbapenem resistance proportions in Gram-negative pathogens from hospital-acquired infections in low- and lower-middle-income countries (2010–2020). Box plots indicate individual study estimates of carbapenem resistance proportions (red dots) and range for first and third quartile. Medians are indicated as a black line and pooled estimates from meta-analysis are displayed as black squares. Whiskers indicate lower and upper end of distribution. Resistance proportions are expressed as percentages (%) of carbapenem-resistant or -non-susceptible isolates among all tested isolates.

The pooled carbapenem resistance proportion was 16.6% (95%CI 10.7-23.4, n=60), 34.9% (95%CI 24.6-45.9, n=50), 37.1% (95%CI 24.6-45.9, n=56), 51.2% (95%CI 27.5-74.7, n=7), 72.4% (95%CI 62.1-81.7%, n=36) for *Escherichia coli, Klebsiella pneumoniae, Pseudomonas aeruginosa, Enterobacter spp., and Acinetobacter baumannii (complex),* respectively (Figure 4).

Furthermore, pooled carbapenem resistance proportions in *A. baumannii (complex)* were significantly lower in WHO Africa (1.6% [95%CI 0.0–10.8%], *n* = 3) compared to WHO South-East Asia (76.8% [95%CI 63.1–88.3%], *n* = 13; *Q* = 57.72, *p* < 0.0001), WHO Eastern Mediterranean (77.0% [95%CI 53.8–94.2%], *n* = 12; *Q* = 32.94, *p* < 0.0001), and WHO West-Pacific (82.8% [95%CI 71.5–81.8%], *n* = 8; *Q* = 74.12, *p* < 0.0001). The pooled proportion of carbapenem-resistant *E. coli* was lower in studies from low-income countries (1.1% [95%CI 0.0–6.9%], *n* = 6) compared to lower-middle-income countries (19.0% [95%CI 12.4–26.4%], *n* = 54; *Q* = 13.65, *p* = 0.002) (Table 2). Pooled proportions of carbapenem resistance in the analysed Gram-negative pathogens were not statistically significantly different between isolates from adults and paediatrics (< 18years).

The pooled proportion of methicillin resistance in *S. aureus* was 48.4% (95% CI 41.7–55.2%, range: 0–100%, median: 57.9%, *n* = 80) (Table 1). No statistically significant differences in MRSA proportions were observed among the four WHO regions (*Q* = 4.93, *p* = 0.1767) and between studies from low-income vs. lower-middle-income countries (*Q* = 0.01, *p* = 0.9208). MRSA proportions were higher in *S. aureus* isolates obtained from ICUs compared to non-ICU wards (54.9% [95%CI 42.7–66.9%] vs. 35.5% [95%CI 236–48.2%], *Q* = 4.75, *p* = 0.0293). In bloodstream HAIs, the pooled MRSA proportion was 36.6% (95% CI 23.4–50.9%, median: 46.7%, *n* = 8) (Table 1). Vancomycin resistance in *S. aureus* isolates from patients with HAI was observed in only 10 out of 39 studies, while no vancomycin-resistant *S. aureus* HAI isolates were found in the remaining studies. The pooled proportion for all 39 studies was 0.6% (95% CI 0.0–1.8%) (Table 1). However, five studies from different WHO regions [16–20] reported a vancomycin resistance proportion higher than 15%. Similarly, only 7 out of 23 studies found vancomycin resistance among MRSA HAI isolates and the pooled resistance proportion for all 23 studies was 1.7% (95%CI 0.0–9.5%). In our study set only one study from Egypt [21] provided data for VRE and reported a resistance proportion of 23%.

A study [22] from Morocco showed that mortality from HAIs due to MRSA and CRAB among adult ICU patients was 80.0% and 52.5%, respectively. Another study from Vietnam [23] conducted in neonatal ICUs reported case fatality rates of 31.8%, 32.5%, and 33.2% for HAIs due to CRAB, CRKP and CRPA, respectively. Among patients with hospital-acquired pneumonia due to CRAB [24] and MRSA [25], all-cause mortality was 60.8% and 66.7%, respectively.

We found a high between-study heterogeneity (*I*^2^ > 80%) in all our analyses. Our set of moderators accounted for 0% (i.e. *R*^2^ = 0%) of the observed heterogeneity in MRSA, less than 5% (TGCR-KP and TGCR-Ent), <20% (CREC, CRPA and CRAB), while they accounted for 44.6% of the observed heterogeneity in TGCR-EC.

## Discussion

Our analysis of 163 studies from L-LMICs showed that pooled resistance proportions of hospital-acquired ESKAPE-E infections to critical antibiotics (range: 16.6–85.5%) are generally high (Table 1). We found cross-regional differences within L-LMICs, notably with the WHO Africa region having a statistically significant lower proportion of TGCR-KP, TGCR-EC, CRAB and CRPA compared to other WHO regions in the L-LMICs (Table 2). The highest antibiotic resistance proportion among Gram-negative ESKAPE-E infections in our study was to third-generation cephalosporins (TGC), with a pooled proportion greater than 75% in *E. coli, Klebsiella pneumoniae and Enterobacter spp*. This is higher than the estimates from HICs (Table 3). The rapid evolution and global spread of extended spectrum beta-lactamases (ESBL) and the simultaneous increase in cephalosporin consumption in L-LMICs may partly explain this difference [4,26,27]. The lower TGCR among *K. pneumoniae* and *E. coli* isolates in the WHO Africa region compared to other regions in the L-LMICs may be due to variation in surveillance capacities, differential access and pattern of antibiotic consumption, among other varying contextual factors [27,28]. Although antimicrobial consumption data in L-LMICs are patchy and difficult to compare [29], available evidence suggests differential consumption of Access and Watch category antibiotics within the L-LMICs [26,30]. As an example, national estimate from Tanzania showed Watch category antibiotics (mainly ceftriaxone) accounted for less than 10% of national consumption [31], while a very high level of TGC consumption was recorded in India [26,30].

We found a high proportion of carbapenem resistance in Gram-negative ESKAPE-E infections in L-LMICs, especially in *A. baumannii* (complex) and *Enterobacter* spp. Compared to other regions, it is similar to carbapenem resistance proportion in China but higher than the proportion recorded among *Acinetobacter* spp. isolates tested in the US, EU/EEA and Japan (Table 3). The expansive multi-drug resistance mechanisms of *A. baumannii*, *P. aeruginosa*, and *K. pneumoniae*, especially the global spread of carbapenemases [32], and a more rapid increase in carbapenem consumption in the L-LMICs [26], are driving increased carbapenem resistance especially in the ICU where they are the leading causes of invasive HAIs [5,26]. Compared to the WHO Africa region, we suspect the higher TGCR we found in other L-LMICs regions, possibly due to increased spread of extended spectrum beta-lactamases (ESBL) and the co-selection of carbapenemases, is driving an increased reliance on carbapenems in these regions [4]. The over-the-counter availability of carbapenems and surge in its consumption has been documented for India, Pakistan and Egypt [4,33]. While data on carbapenem consumption is very scarce in WHO Africa, available evidence suggests that access and use of carbapenems is very low for various reasons including its absence over-the-counter, and on the essential medicine list of many African countries before 2020 [31,34,35]. Other factors like underestimation due to limited diagnostic stewardship and surveillance as well as varying infection prevention and control (IPC) capacity, may also contribute to the differences observed within L-LMICs and when compared to HICs [36].

The proportion of MRSA in our study is similar to Japan (46.1%), the United States (40.6%) and the regional estimates from South America (47.7%), but lower than the national estimates from South Korea (69.4%) [37] (Table 3). It is however higher than the national estimate from other upper-middle and high-income regions like China and the Europe (Table 3). Our analysis showed no significant difference in MRSA proportions across the four WHO regions and even when disaggregated by national income levels. In line with previous studies from high-income settings, MRSA is prominent in the ICU as the proportion of MRSA in our study was statistically significantly higher in the ICU relative to the non-ICU wards [38]. These findings point to the pervasive nature of MRSA globally in hospital settings despite being the most frequent HAI targeted for IPC interventions [36]. MRSA in bloodstream infections is an important indicator for monitoring the progress towards the attainment of the Sustainable Development Goals (target 3.d.2). Our results showed a somewhat higher median proportion of MRSA in bloodstreams infections (46.7%) compared to a median proportion of 33.3% and 15% reported in the 2021 WHO GLASS report for low and middle-income countries (LMICs) and HICs, respectively [27]. It is noteworthy that our study included only data from patients with HAIs while the data submitted to GLASS included both HAIs and community-acquired infections [27,39]. Similar to findings from a recent review [40], our results showed MRSA remains generally susceptible to vancomycin. However, the pooled prevalence of 1.7% is higher than estimates (≤ 0.1%) from upper-middle and high-income countries like the United States, Japan, China and South Korea (Table 3) [37,41–43].

Similar to other reviews on hospital-acquired infections and associated antibiotic resistances [5,44–46], we found a large variance across the individual study estimates. Therefore, other variables not determinable from our data account for the large heterogeneity. A complex interwoven of biologic, socio-demographic, economic, political and environmental factors, with regional and local peculiarities, are known to influence the emergence and spread of AMR [47]. In addition, variability in study settings, sample selection, methodological differences in pathogen identification and AST, surveillance and antibiotic stewardship may also explain the high heterogeneity. Following revisions since 2010, it is possible some studies applied outdated EUCAST and CLSI carbapenem breakpoints that might have underestimated carbapenem resistance in *Enterobacteriaceae* [48]. The varying AMR patterns we found in HAIs among regions underline the need for local surveillance to better understand the observed differences, and in turn, develop appropriate recommendations on antibiotic use and antibiotic stewardship measures.

To our knowledge, our study represents the first systematic review of resistance patterns in HAIs from the L-LMICs. The majority of studies were based on routine HAI surveillance data, including data from unselected patient cohorts treated in hospitals. However, we noted some limitations in our study. Firstly, despite including a large number of studies, our data covered only 24 out of 82 L-LMICs with limited national and regional representativeness. This potentially limits the external validity of our results to some extent. Nevertheless, the overall quality of the studies and thus, the quality of evidence, was moderate to high. Secondly, there is a general dearth of data on VRE, and a relatively low number of studies were available from countries in the Western Pacific WHO region and for some pathogens from WHO Africa, which limits the robustness of the evidence of certain AMR patterns in these regions. Therefore, our pooled effect estimates should be interpreted with caution.

In conclusion, the high resistance of clinically important bacterial infections to important antibiotics highlights its potential impact on health systems and livelihoods in resource-constrained regions that already suffer from wider socio-economic challenges. While the overarching strategies to combat AMR are well described at the global level [49], the varying regional AMR patterns we found suggest the need for priorities to be redirected to increased understanding of AMR dynamics at the regional and local levels and the use of such evidence to tailor sustainable solutions.

## Supplementary Material

Supplemental Material
