# Supplementary material for: Antibiotic resistance in hospital-acquired ESKAPE-E infections in low- and lower-middle-income countries: a systematic review and meta-analysis
Source: Emerg Microbes Infect. 2022 Feb 4;11(1):443–51. doi: 10.1080/22221751.2022.2030196 (PMC8820817; doi:10.1080/22221751.2022.2030196)
Supplement: Supplemental Material [file TEMI_A_2030196_SM3719.zip › Suppl files/Supplementary_Table.docx]

Supplementary Document

Table of Content

1. Search string………………………………………………………………………………...Pages 2-5
2. Characteristics of included studies………………………………………………………… Pages 6-12
3. Risk of bias assessment table……………………………………………………………… Pages 13-16
4. References of included studies………………………………………………………….…. Pages 17-26

Appendix 1 Search string 2

1.1 EMBASE and Web of Science

All fields:

(“enterococcus faecium“ OR “E. faecium“ OR “enterococc* “ OR “Glycopeptide-resistant enterococcus faecium“ OR “Glycopeptide-resistant E. faecium“ OR “Glycopeptide-resistant enterococc* “ OR “vancomycin-resistant enterococcus faecium“ OR “vancomycin-resistant E. faecium“ OR “vancomycin-resistant enterococc* “ OR “Glycopeptide resistant enterococcus faecium“ OR “Glycopeptide resistant E. faecium“ OR “Glycopeptide resistant enterococc* “ OR “vancomycin resistant enterococcus faecium“ OR “vancomycin resistant E. faecium“ OR “vancomycin resistant enterococc* “ OR “VREF“ OR “VRE“ OR “multi-drug resistant enterococcus faecium” OR “multi-drug resistant E. faecium” OR “multi-drug resistant enterococc*” OR “multidrug resistant enterococcus faecium” OR “multidrug resistant E. faecium” OR “multidrug resistant enterococc*” OR “MDR enterococcus faecium” OR “MDR E. faecium” OR “MDR enterococc*” OR “extensively-drug resistant enterococcus faecium” OR “extensively-drug resistant E. faecium” OR “extensively-drug resistant enterococc*” OR “XDR enterococcus faecium” OR “XDR E. faecium” OR “XDR enterococc*” OR “pan-drug resistant enterococcus faecium” OR “pan-drug resistant E. faecium” OR “pan-drug resistant enterococc*” OR “pandrug resistant enterococcus faecium” OR “pandrug resistant E. faecium” OR “pandrug resistant enterococc*” OR “PDR enterococcus faecium” OR “PDR E. faecium” OR “PDR enterococc*” OR “staphylococcus aureus” OR “S.aureus “ OR “staphylococc* “ OR “Glycopeptide-resistant staphylococcus aureus“ OR “Glycopeptide-resistant S. aureus“ OR “Glycopeptide-resistant staphylococc* “ OR “vancomycin-resistant staphylococcus aureus“ OR “vancomycin-resistant S. aureus“ OR “vancomycin-resistant staphylococc* “ OR “Beta-lactams-resistant staphylococcus aureus“ OR “Beta-lactams-resistant S. aureus“ OR “Beta-lactams-resistant staphylococc* “ OR “β-lactams-resistant staphylococcus aureus“ OR “β-lactams-resistant S. aureus“ OR “β-lactams-resistant staphylococc* “ OR “Methicillin-resistant staphylococcus aureus“ OR “Methicillin-resistant S. aureus“ OR “Methicillin-resistant staphylococc* “ OR “Glycopeptide resistant staphylococcus aureus“ OR “Glycopeptide resistant S. aureus“ OR “Glycopeptide resistant staphylococc* “ OR “vancomycin resistant staphylococcus aureus“ OR “vancomycin resistant S. aureus“ OR “vancomycin resistant staphylococc* “ OR “Beta-lactams resistant staphylococcus aureus“ OR “Beta-lactams resistant S. aureus“ OR “Beta-lactams resistant staphylococc* “ OR “β-lactams resistant staphylococcus aureus“ OR “β-lactams resistant S. aureus“ OR “β-lactams resistant staphylococc* “ OR “Methicillin resistant staphylococcus aureus“ OR “Methicillin-resistant S. aureus“ OR “Methicillin resistant staphylococc* “ OR “MRSA“ OR “VRSA“ OR “multi-drug resistant staphylococcus aureus” OR “multi-drug resistant S. aureus” OR “multi-drug resistant staphylococc*” OR “multidrug resistant staphylococcus aureus” OR “multidrug resistant s. aureus” OR “multidrug resistant staphylococc*” OR “MDR staphylococcus aureus” OR “MDR S. aureus” OR “MDR staphylococc*” OR “extensively-drug resistant staphylococcus aureus” OR “extensively-drug resistant S. aureus” OR “extensively-drug resistant staphylococc*” OR “XDR staphylococcus aureus” OR “XDR S. aureus” OR “XDR staphylococc*” OR “pan-drug resistant staphylococcus aureus” OR “pan-drug resistant S. aureus” OR “pan-drug resistant staphylococc*” OR “pandrug resistant staphylococcus aureus” OR “pandrug resistant S. aureus” OR “pandrug resistant staphylococc*” OR “PDR staphylococcus aureus” OR “PDR S. aureus” OR “PDR staphylococc*” OR “klebsiella pneumoniae“ OR “K. pneumoniae“ OR “klebsiell* “ OR “Carbapenem-resistant klebsiella pneumoniae“ OR “Carbapenem-resistant K. pneumoniae“ OR “Carbapenem-resistant klebsiella “ OR “Carbapenem resistant klebsiella pneumoniae“ OR “Carbapenem resistant K. pneumoniae “ OR “Carbapenem resistant klebsiella “ OR “Beta-lactams resistant klebsiella pneumoniae“ OR “Beta-lactams resistant K. pneumoniae“ OR “Beta-lactams resistant klebsiella“ OR “β-lactams resistant klebsiella pneumoniae“ OR “β-lactams resistant K. pneumoniae“ OR “β-lactams resistant klebsiella“ OR “Beta-lactams-resistant klebsiella pneumoniae“ OR “Beta-lactams-resistant K. pneumoniae“ OR “Beta-lactams-resistant klebsiella“ OR “β-lactams-resistant klebsiella pneumoniae“ OR “β-lactams-resistant K. pneumoniae“ OR “β-lactams-resistant klebsiella“ OR “3rd generation cephalosporin-resistant klebsiella pneumoniae“ OR “3rd generation cephalosporin-resistant K. pneumoniae“ OR “3rd generation cephalosporin-resistant klebsiella “ OR “3rd generation cephalosporin resistant klebsiella pneumoniae“ OR “3rd generation cephalosporin resistant K. pneumoniae“ OR “3rd generation cephalosporin resistant klebsiella“ OR “third generation cephalosporin-resistant klebsiella pneumoniae“ OR “third generation cephalosporin-resistant K. pneumoniae“ OR “third generation cephalosporin-resistant klebsiella “ OR “third generation cephalosporin resistant klebsiella pneumoniae“ OR “third generation cephalosporin resistant K. pneumoniae“ OR “third generation cephalosporin resistant klebsiella“ OR “CRKP“ OR “multi-drug resistant klebsiella pneumoniae” OR “multi-drug resistant K. pneumoniae” OR “multi-drug resistant klebsiella” OR “multidrug resistant klebsiella pneumoniae” OR “multidrug resistant K. pneumoniae” OR “multidrug resistant klebsiella” OR “MDR klebsiella pneumoniae” OR “MDR K. pneumoniae” OR “MDR klebsiella” OR “extensively-drug resistant klebsiella pneumoniae” OR “extensively-drug resistant K. pneumoniae” OR “extensively-drug resistant klebsiella” OR “XDR klebsiella pneumoniae” OR “XDR K. pneumoniae” OR “XDR klebsiella” OR “pan-drug resistant klebsiella pneumoniae” OR “pan-drug resistant K. pneumoniae” OR “pan-drug resistant klebsiella” OR “pandrug resistant klebsiella pneumoniae” OR “pandrug resistant K. pneumoniae” OR “pandrug resistant klebsiella” OR “PDR klebsiella pneumoniae” OR “PDR K. pneumoniae” OR “PDR klebsiella” OR “acinetobacter baumannii“ OR “A. baumannii“ OR “Carbapenem-resistant acinetobacter baumannii“ OR “Carbapenem-resistant A. baumannii“ OR “Carbapenem-resistant acinetobacter“ OR “Carbapenem resistant acinetobacter baumannii“ OR “Carbapenem resistant A. baumannii“ OR “Carbapenem resistant acinetobacter“ OR “CRAB“ OR “multi-drug resistant acinetobacter baumannii” OR “multi-drug resistant A. baumannii” OR “multi-drug resistant acinetobacter” OR “multidrug resistant acinetobacter baumannii” OR “multidrug resistant A. baumannii” OR “multidrug resistant acinetobacter” OR “MDR acinetobacter baumannii” OR “MDR A. baumannii” OR “MDR acinetobacter” OR “extensively-drug resistant acinetobacter baumannii” OR “extensively-drug resistant A. baumannii” OR “extensively-drug resistant acinetobacter” OR “XDR acinetobacter baumannii” OR “XDR A. baumannii” OR “XDR acinetobacter” OR “pan-drug resistant acinetobacter baumannii” OR “pan-drug resistant A. baumannii” OR “pan-drug resistant acinetobacter” OR “pandrug resistant acinetobacter baumannii” OR “pandrug resistant A. baumannii” OR “pandrug resistant acinetobacter” OR “PDR acinetobacter baumannii” OR “PDR A. baumannii” OR “PDR acinetobacter” OR “pseudomonas aeruginosa“ OR “P. aeruginosa“ OR “Carbapenem-resistant pseudomonas aeruginosa“ OR “Carbapenem-resistant P. aeruginosa“ OR “Carbapenem-resistant pseudomonas“ OR “Carbapenem resistant pseudomonas aeruginosa“ OR “Carbapenem resistant P. aeruginosa“ OR “Carbapenem resistant pseudomonas“ OR “CRPA“ OR “multi-drug resistant pseudomonas aeruginosa” OR “multi-drug resistant P. aeruginosa” OR “multi-drug resistant pseudomonas” OR “multidrug resistant pseudomonas aeruginosa” OR “multidrug resistant P. aeruginosa” OR “multidrug resistant pseudomonas” OR “MDR pseudomonas aeruginosa” OR “MDR P. aeruginosa” OR “MDR pseudomonas” OR “extensively-drug resistant pseudomonas aeruginosa” OR “extensively-drug resistant P. aeruginosa” OR “extensively-drug resistant pseudomonas” OR “XDR pseudomonas aeruginosa” OR “XDR P. aeruginosa” OR “XDR pseudomonas” OR “pan-drug resistant pseudomonas aeruginosa” OR “pan-drug resistant P. aeruginosa” OR “pan-drug resistant pseudomonas” OR “pandrug resistant pseudomonas aeruginosa” OR “pandrug resistant P. aeruginosa” OR “pandrug resistant pseudomonas” OR “PDR pseudomonas aeruginosa” OR “PDR P. aeruginosa” OR “PDR pseudomonas” OR “Enterobacter species“ OR “Enterobacter spp.“ OR “Carbapenem-resistant Enterobacter species“ OR “Carbapenem-resistant Enterobacter spp.“ OR “Carbapenem-resistant enterobacter“ OR “Carbapenem resistant Enterobacter species“ OR “Carbapenem resistant Enterobacter spp.“ OR “Carbapenem resistant enterobacter“ OR “Beta-lactams resistant Enterobacter species“ OR “Beta-lactams resistant Enterobacter spp.“ OR “Beta-lactams resistant enterobacter“ OR “β-lactams resistant Enterobacter species“ OR “β-lactams resistant Enterobacter spp.“ OR “β-lactams resistant enterobacter“ OR “Beta-lactams resistant-Enterobacter species“ OR “Beta-lactams-resistant Enterobacter spp.“ OR “Beta-lactams-resistant enterobacter“ OR “β-lactams-resistant Enterobacter species“ OR “β-lactams-resistant Enterobacter spp.“ OR “β-lactams-resistant enterobacter“ OR “3rd generation cephalosporin-resistant Enterobacter species“ OR “3rd generation cephalosporin-resistant Enterobacter spp.“ OR “3rd generation cephalosporin-resistant enterobacter“ OR “3rd generation cephalosporin resistant Enterobacter species“ OR “3rd generation cephalosporin resistant Enterobacter spp.“ OR “3rd generation cephalosporin resistant enterobacter“ OR “third generation cephalosporin-resistant Enterobacter species“ OR “third generation cephalosporin-resistant Enterobacter spp.“ OR “third generation cephalosporin-resistant enterobacter“ OR “third generation cephalosporin resistant Enterobacter species“ OR “third generation cephalosporin resistant Enterobacter spp.“ OR “third generation cephalosporin resistant enterobacter“ OR “multi-drug resistant Enterobacter species” OR “multi-drug resistant Enterobacter” OR “multidrug resistant Enterobacter species” OR “multidrug resistant Enterobacter” OR “MDR Enterobacter species” OR “MDR Enterobacter” OR “extensively-drug resistant Enterobacter species” OR “extensively-drug resistant Enterobacter” OR “XDR Enterobacter species” OR “XDR Enterobacter” OR “pan-drug resistant Enterobacter species” OR “pan-drug resistant Enterobacter” OR “pandrug resistant Enterobacter species” OR “pandrug resistant Enterobacter” OR “PDR Enterobacter species” OR “PDR Enterobacter” OR “escherichia coli“ OR “E. coli“ OR “Carbapenem-resistant escherichia coli“ OR “Carbapenem-resistant E. coli“ OR “Carbapenem-resistant escherichia“ OR “Carbapenem resistant escherichia coli“ OR “Carbapenem resistant E. coli“ OR “Carbapenem resistant escherichia“ OR “Beta-lactams resistant escherichia coli“ OR “Beta-lactams resistant E. coli“ OR “Beta-lactams resistant escherichia“ OR “β-lactams resistant escherichia coli“ OR “β-lactams resistant E. coli“ OR “β-lactams resistant escherichia“ OR “Beta-lactams resistant escherichia coli“ OR “Beta-lactams-resistant E. coli“ OR “Beta-lactams-resistant escherichia“ OR “β-lactams-resistant escherichia coli“ OR “β-lactams-resistant E. coli“ OR “β-lactams-resistant escherichia“ OR “3rd generation cephalosporin-resistant escherichia coli“ OR “3rd generation cephalosporin-resistant E. coli“ OR “3rd generation cephalosporin-resistant escherichia“ OR “3rd generation cephalosporin resistant escherichia coli“ OR “3rd generation cephalosporin resistant E. coli“ OR “3rd generation cephalosporin resistant escherichia“ OR “third generation cephalosporin-resistant escherichia coli“ OR “third generation cephalosporin-resistant E. coli“ OR “third generation cephalosporin-resistant escherichia“ OR “third generation cephalosporin resistant escherichia coli“ OR “third generation cephalosporin resistant E. coli“ OR “third generation cephalosporin resistant escherichia“ OR “CREC“ OR “multi-drug resistant escherichia coli” OR “multi-drug resistant E. coli” OR “multi-drug resistant escherichia” OR “multidrug resistant escherichia coli” OR “multidrug resistant E. coli” OR “multidrug resistant escherichia” OR “MDR escherichia coli” OR “MDR E. coli” OR “MDR escherichia” OR “extensively-drug resistant escherichia coli” OR “extensively-drug resistant E. coli” OR “extensively-drug resistant escherichia” OR “XDR escherichia coli” OR “XDR E. coli” OR “XDR escherichia” OR “pan-drug resistant escherichia coli” OR “pan-drug resistant E. coli” OR “pan-drug resistant escherichia” OR “pandrug resistant escherichia coli” OR “pandrug resistant E. coli” OR “pandrug resistant escherichia” OR “PDR escherichia coli” OR “PDR E. coli” OR “PDR escherichia”)

AND

(“resistance proportion* “ OR “resistance rate* “ OR “proportion* “ OR “rate* “ OR “antimicrobial resistanc* “ OR “prevalence“ OR “incidence“ OR “burden“ OR “epidemiological data“ OR “epidemiolog* “ OR “frequenc* “ OR “surveillance“ OR “health survey“ OR “resistance profile”)

AND

(“Afghanistan“ OR “Guinea-Bissau“ OR “Sierra Leone“ OR “Burkina Faso“ OR “Haiti“ OR “Somalia“ OR “Burundi“ OR “Korea, Dem. Peoples Rep.“ OR “North Korea“ OR “South Sudan“ OR “Central African Republic“ OR “Central Africa“ OR “Liberia“ OR “Sudan“ OR “Chad“ OR “Madagascar“ OR “Syrian Arab Republic“ OR “Syria“ OR “Congo, Dem. Rep“ OR “Congo“ OR “Malawi“ OR “Tajikistan“ OR “Eritrea“ OR “Mali“ OR “Togo“ OR “Ethiopia“ OR “Mozambique“ OR “Uganda“ OR “Gambia“ OR “Niger“ OR “Yemen“ OR “Guinea“ OR “Rwanda“ OR “Angola“ OR “Bangladesh“ OR “Benin“ OR “Bhutan“ OR “Bolivia“ OR “Cabo Verde“ OR “Cambodia“ OR “Cameroon“ OR “Comoros“ OR “Congo, Rep.“ OR “Côte d Ivoire“ OR “Ivory Coast“ OR “Djibouti“ OR “Egypt, Arab Rep.“ OR “Egypt“ OR “El Salvador“ OR “Eswatini“ OR “Ghana“ OR “Honduras“ OR “India“ OR “Kenya“ OR “Kiribati“ OR “Kyrgyz Republic“ OR “Lao PDR“ OR “Lao“ OR “Lesotho“ OR “Mauritania“ OR “Micronesia, Fed. Sts.“ OR “Micronesia“ OR “Moldova“ OR “Mongolia“ OR “Morocco“ OR “Myanmar“ OR “Nepal“ OR “Nicaragua“ OR “Nigeria“ OR “Pakistan“ OR “Papua New Guinea“ OR “Philippines“ OR “São Tomé and Principe“ OR “Senegal“ OR “Solomon Islands“ OR “Sri Lanka“ OR “Tanzania“ OR “Timor-Leste“ OR “Tunisia“ OR “Ukraine“ OR “Uzbekistan“ OR “Vanuatu“ OR “Vietnam“ OR “West Bank and Gaza“ OR “West Bank“ OR “Gaza“ OR “Zambia“ OR “Zimbabwe“ OR “Asia“ OR “Africa“ OR “Latin America“ OR “Caribbean“ OR “low income“ OR “lower middle income“ OR “developing countr* “ OR “low resource“ OR “low-income“ OR “lower-middle income“ OR “low-resource“ OR “resource-limite* “)

AND

(“healthcare associated infection“ OR “healthcare acquired infection” OR “hospital infection“ OR “nosocomial“ OR “healthcare-associated infection“ OR “healthcare-acquired infection“ OR “hospital acquired“ OR “hospital associated“ OR “intensive care unit acquired infection“ OR “ICU-acquired” OR ”ICU acquired” OR “HAI* “ OR “catheter-related” OR “catheter-associated” OR “ventilator-related” OR “ventilator-associated” OR “central line-associated” OR “central line-related” OR “surgical site infection”)

AND

[2010-2020]/py

AND

([article]/lim

1.2 Global Index Medicus

(Escherichia OR enterococcus OR Staphylococcus OR Klebsiella OR Acinetobacter OR Pseudomonas OR Enterobacter)

AND

(epidemiology OR surveillance OR resistance OR proportion OR burden OR incidence OR prevalence)

AND

(nosocomial OR hospital-acquired OR healthcare-associated)

AND

[2010-2020]/py

Language restriction: English, Spanish, Portuguese, French

Table 1: Characteristics of included studies ([1-163])

| **Study** | **Country** | **Income** | **WHO region** | **Patient age group** | **Hospital ward** | **Drug-bug combination** |
| --- | --- | --- | --- | --- | --- | --- |
| Abdallah 2014 | Egypt | LMI | EMR | Adults | ICU | MRSA |
| Abd-Elmonsef 2018 | Egypt | LMI | EMR | Adults | ICU | MRSA, VSA |
| Abdoulaye 2018 | Niger | LMI | AFR | Mixed | Surgical ward  (non-ICU) | MRSA, CREC, CRPA,  CephEC |
| Abdulall 2018 | Egypt | LMI | EMR | Not reported | ICU | CephKP, CRKP |
| Afroz 2017 | Bangladesh | LMI | SEAR | Mixed | Hospital | CRKP, CephKP |
| Agmy 2013 | Egypt | LMI | EMR | Adults | Non-ICU | MRSA, VMRSA, VSA |
| Ahmad 2014 | Pakistan | LMI | EMR | Mixed | Non-ICU | MRSA |
| Ahmad 2018 | Syria | LI | EMR | Mixed | Department of general surgery  (non-ICU) | CRPA, CREC,  CephEC |
| Ahmed 2014 | Egypt | LMI | EMR | Mixed | Department of general surgery  (non-ICU) | MRSA |
| Fakhr 2018 | Egypt | LMI | EMR | Mixed | Hospital | MRSA, CRPA, VSA, CREC,  CephEC |
| Ahoyo 2014 | Benin | LMI | AFR | Mixed | Hospital | MRSA, VSA, CREC, CRPA, CRABC |
| Alemayehu 2019 | Ethiopia | LI | AFR | Infants + Children | Hospital | MRSA |
| Alioua 2014 | Algeria | LMI | EMR | Mixed | Hospital | MRSA |
| Amutha 2015 | India | LMI | SEAR | Adults | Hospital | VSA, MRSA |
| Anago 2015 | Benin | LMI | AFR | Not reported | Hospital | CephEC, CREC |
| Andrianarivelo 2017 | Madagascar | LI | AFR | Mixed | Hospital | MRSA,  VSA,  VMRSA |
| Anuradha 2014 | India | LMI | SEAR | Mixed | Hospital | CREC, CRPA, MRSA, VSA, CRKP, CephKP |
| Arya 2014 | India | LMI | SEAR | Mixed | Hospital | CREC |
| Ateba 2013 | Cameroon | LMI | SEAR | Mixed | Hospital | CRPA |
| Awoke 2019 | Ethiopia | LI | AFR | Not reported | Hospital | CephEC |
| Azzab 2016 | Egypt | LMI | EMR | Mixed | Emergency ICU | VSA |
| Babu 2011a | India | LMI | SEAR | Not reported | ICU | CephKP, CRKP, CRAB, CRPA |
| Babu 2001b | India | LMI | SEAR | Mixed | Hospital | CRPA |
| Bammigatti 2017 | India | LMI | SEAR | Mixed | Medical ICU | CRAB, CRPA, CREC,  CephEC, CephKP, CRKP |
| Bashir 2019 | NIgeria | LMI | AFR | Adults | Hospital | CephEC, CREC, CephKP, CRKP |
| Bhadade 2017 | India | LMI | SEAR | Adults | Medical ICU | CREC,  CephEC, CRKP, CephKP, CRPA, VSA, MRSA |
| Bhatia 2017 | India | LMI | SEAR | Mixed | ICU | CRAB, CRKP, CephKP, CRPA |
| Bhattacharya 2016 | India | LMI | SEAR | MIxed | Hospital | VSA, MRSA, VMRSA |
| Biedenbach 2016 | Vietnam | LMI | WPR | Not reported | Hospital | CRAB, CRPA |
| Bijapur 2015 | India | LMI | SEAR | Mixed | Hospital | CREC,  CephEC |
| Bishwas 2020 | India | LMI | SEAR | Adults | Medical ICU | CRAB |
| Chaari 2013 | Tunisia | LMI | EMR | Adults | Medical-surgical ICU | CRAB |
| Chandrasekaran 2016 | India | LMI | SEAR | Not reported | Hospital | CREC,  CephEC,  CRPA,  CRKP,  CephKP,  MRSA,  VSA |
| Chaudhury 2016 | India | LMI | SEAR | Not reported | ICU | MRSA,  CREC,  CephEC,  CREnt  CephEnt,  VSA |
| Chavan 2017 | India | LMI | SEAR | Not reported | Mixed, surgical wards and ICU | CREC,  CephEC, |
| Rosenthal 2020 | India | LMI | SEAR | Mixed | ICU | CREC,  CephEC  MRSA,  CRKP,  CephKP |
| Datta 2014 | India | LMI | SEAR | Mixed | ICU | CRPA,  CRKP,  CephKP,  CREC,  CephEC,  MRSA,  VSA |
| Dayyab 2018 | Nigeria | LMI | AFR | Adults | Hospital | MRSA,  CephEC,  CephKP |
| Doddamani 2013 | India | LMI | SEAR | Mixed | Hospital | VSA,  MRSA |
| Dubey 2013a | India | LMI | SEAR | Mixed | Hospital | MRSA,  VMRSA |
| El-Kholy 2012 | Egypt | LMI | EMR | Mixed | ICU | CRAB,  CRPA |
| El-Mahdy 2019 | Egypt | LMI | EMR | Mixed | Hospital | CRPA |
| El-Mahdy 2018 | Egypt | LMI | EMR | Mixed | Medical and surgical ICUs | CRKP,  CephKP, |
| El Mekes 2020 | Morocco | LMI | EMR | Adults | Medical and surgical ICUs | MRSA,  CRAB |
| El-Nawawy 2019 | Egypt | LMI | EMR | Infants+Children | PICU | CRKP,  CephKP |
| El-Sahrigy 2019 | Egypt | LMI | EMR | Infants+Children | PICU | VSA |
| Elmouaden 2019 | Morocco | LMI | EMR | Not reported | Hospital | CRPA |
| Eman 2018 | Egypt | LMI | EMR | Mixed | ICU | CRAB |
| Hammad 2011 | Egypt | LMI | EMR | Mixed | Neurlogical ICU | CREC,  CRKP,  CephKP,  CRPA,  CephEC |
| Fatima 2013 | Pakistan | LMI | EMR | Mixed | Obstetrics and gynaecology unit (non-ICU) | MRSA,  VSA |
| Feleke 2018 | Ethiopia | LI | AFR | Mixed | Hospital | MRSA,  CephEC |
| Ferjani 2015 | Tunisia | LMI | EMR | Mixed | Hospital | CephEC,  CREC |
| Fouad 2013 | Egypt | LMI | EMR | Mixed | ICU | CRAB |
| Fox-Lewis 2018 | Cambodia | LMI | WPR | Infants+children | Pediatric hospital | MRSA,  CephKP,  CRKP,  CRAB,  CRPA,  CephEC,  CREC |
| Gaber 2020 | Egypt | LMI | EMR | Mixed | Hospital | CRPA |
| Gashaw 2018 | Ethiopia | LI | AFR | Mixed | Hospital | MRSA,  CephEC,  CREC |
| Golia 2013 | India | LMI | SEAR | Mixed | ICU | CRPA,  CephEC,  CREC |
| Gupta 2018 | India | LMI | SEAR | Mixed | Hospital | CephKP,  CRKP,  CRAB,  CRPA |
| Gupta 2017 | India | LMI | SEAR | Mixed | Medical and surgical ICU | CRKP,  CephKP,  CRAB,  CRPA |
| Hashem 2017 | Egypt | LMI | EMR | Mixed | ICU | MRSA,  VSA,  VMRSA |
| Hassan 2017 | Egypt | LMI | EMR | Infants+children | Pediatric hospital | MRSA |
| Hassuna 2020 | Egypt | LMI | EMR | Infants | NICU | CRKP,  CephKP |
| Hassuna 2016 | Egypt | LMI | EMR | Mixed | Hospital | CRPA |
| Hope 2019 | Uganda | LI | AFR | Mixed | Hospital | MRSA |
| Hossain 2014 | Bangladesh | LMI | SEAR | Mixed | Hospital | CephEC,  CREC |
| Iliyasu 2020 | Nigeria | LMI | AFR | Mixed | Hospital | MRSA |
| Iliyasu 2016 | Nigeria | LMI | AFR | Mixed | ICU | CephKP |
| Iliyasu 2018 | Nigeria | LMI | AFR | Mixed | Hospital | MRSA,  CephKP,  CephEC,  CREC |
| Jadhav 2020 | India | LMI | SEAR | Mixed | ICU | CRABC,  CRPA |
| Jain 2017 | India | LMI | SEAR | Mixed | Hospital | MRSA,  VSA |
| Jyoti 2016 | India | LMI | SEAR | Mixed | Hospital | MRSA,  VMRSA,  VSA |
| Kahsay 2014 | Ethiopia | LI | AFR | Adults | Hospital | MRSA,  VSA,  VMRSA |
| Kalayu 2019 | Ethiopia | LI | AFR | Mixed | Hospital | MRSA,  VSA,  CephEC,  CREC |
| Khairy 2020 | Egypt | LMI | EMR | Mixed | Hospital | CRKP,  CephKP |
| Khalid 2018 | Pakistan | LMI | EMR | Mixed | Mixed, department of orthopedic surgery | MRSA |
| Khan 2017 | India | LMI | SEAR | Mixed | ICU | CRKP,  CREC,  CRPA,  CRAB,  CephEC,  CephKP |
| Khatun 2015 | Bangladesh | LMI | SEAR | Mixed | ICU | CRAB |
| Khurana 2017 | India | LMI | SEAR | Mixed | ICU | CRPA |
| Kishk 2020 | Egypt | LMI | EMR | Mixed | Hospital | CRPA |
| Kotb 2020 | Egypt | LMI | EMR | Mixed | ICU | CREC,  CREnt |
| Kumar 2018a | India | LMI | SEAR | Mixed | Hospital | CRAB,  CREC,  CephEC,  CRKP,  CephKP |
| Labi 2019 | Ghana | LMI | AFR | Mixed | Hospital | MRSA |
| Labib 2018 | Egypt | LMI | EMR | Infants+children | PICUs | CRPA,  CRKP,  CephKP,  CREC,  CephEC,  CREnt, CephEnt |
| Lachhab 2017 | Morocco | LMI | EMR | Adults | Medical and surgical ICU | CRAB,  CRKP,  CephKP |
| Lakoh 2020 | Sierra Leone | LI | AFR | Adults | Hospital | CREC,  CephEC,  CRKP,  CephKP,  CRAB |
| Le 2016 | Vietnam | LMI | WPR | Infants+children | PICU | CRKP,  CephKP,  CRPA,  CRAB,  MRSA,  CREnt,  CephEC |
| Mahfoud 2015 | Syria | LI | EMR | Mixed | ICU | CRPA |
| Esmat 2018 | Egypt | LMI | EMR | Mixed | Hospital | CREC,  CephEC,  CRPA,  MRSA,  VSA |
| Soliman 2018 | Egypt | LMI | EMR | Mixed | ICU | CRAB |
| Manjhi 2018 | India | LMI | SEAR | Infants+children | PICU | CRPA,  CRKP,  CephKP,  CREC,  CephEC |
| Maoulainine 2014 | Morocco | LMI | EMR | Infants | NICU | CRKP |
| Azab 2015 | Egypt | LMI | EMR | Not reported | Hospital | CRPA |
| Mawalla 2011 | Tanzania | LMI | AFR | Mixed | Surgical wards (non-ICU) | MRSA |
| Mehta 2014 | India | LMI | SEAR | Mixed | Hospital | MRSA,  VSA,  VMRSA,  CREC,  CephEC,  CRKP,  CephKP |
| Mitharwal 2016 | India | LMI | SEAR | Mixed | Medical and surgical ICU | CRPA,  MRSA |
| Moemen 2015 | Egypt | LMI | EMR | Mixed | ICU | VRE |
| Mohanty 2017 | India | LMI | SEAR | Adults | Hospital | VSA,  CREC,  CephEC,  CRKP,  CephKP |
| Moolchandani 2017 | India | LMI | SEAR | Mixed | ICUs, incl PICU and NICU | CREC,  CephEC,  CREnt,  CephEnt,  MRSA,  VSA,  VMRSA |
| Motbainor 2020 | Ethiopia | LI | AFR | Mixed | Hospital | CRPA |
| Mundhada 2015 | India | LMI | SEAR | Mixed | Department of orthopedics, surgery and obstetrics and gynecology (non-ICU) | MRSA,  CephEC |
| Murphy 2016 | Nigeria | LMI | AFR | Mixed | Hospital | MRSA,  CREC,  CephEC,  CRPA,  CephEnt,  CREnt |
| Narula 2020 | India | LMI | SEAR | Mixed | Department of general surgery (non-ICU) | MRSA,  VSA,  VMRSA,  CRKP,  CephKP,  CRPA,  CREC,  CephEC |
| Negi 2015 | India | LMI | SEAR | Adults | Non-ICU | MRSA,  VSA,  VMRSA,  CREC,  CephEC,  CRPA |
| Niranjan 2011 | India | LMI | SEAR | Mixed | ICUs, including NICU, PICU and adult ICU | CRKP,  CephKP,  CREC |
| Njoku 2019 | Nigeria | LMI | AFR | Adults | Department of obstetrics and gynaecology (non-ICU) | MRSA,  CREC,  CephEC,  CRKP,  CephKP |
| Nwankwo 2014 | Nigeria | LMI | AFR | Mixed | Surgical wards (non-ICU) | MRSA,  CephEC,  CephKP |
| Olajubu 2012 | Nigeria | LMI | AFR | Mixed | Hospital | CephEC,  CephKP |
| Padmavathy 2018 | India | LMI | SEAR | Mixed | Hospital | MRSA |
| Padmini 2019 | India | LMI | SEAR | Mixed | Hospital | CREC,  CephEC,  CRKP,  CephKP |
| Pal 2019 | India | LMI | SEAR | Adults | Surgical wards (non-ICU) | MRSA,  VSA,  VMRSA |
| Parajuli 2017 | Nepal | LMI | SEAR | Mixed | ICU | CRPA,  CREC,  CephEC |
| Pathak 2014 | India | LMI | SEAR | Mixed | Surgical wards (non-ICU) | MRSA,  VSA |
| Patil 2015 | India | LMI | SEAR | Mixed | Hospital | MRSA,  VSA,  CREC,  CREC,  CephEC,  CRPA |
| Peters 2019 | Vietnam | LMI | WPR | Infants | NICU | CRAB,  CRKP,  CRPA |
| Prakash 2013 | India | LMI | SEAR | Not reported | Hospital | CREC,  CephEC |
| Prakash 2017 | India | LMI | SEAR | Mixed | Hospital, | MRSA |
| Rafai 2015 | Central African Republic | LI | AFR | Mixed | Non-ICU | MRSA,  CRAB,  CephEnt,  CephKP,  CephEC |
| Rafiq 2019 | Pakistan | LMI | EMR | Adults | Department of orthopedic surgery (non-ICU) | MRSA |
| Ramakrishnan 2019 | India | LMI | SEAR | Adults | Hospital | CREC,  CephEC,  CRKP,  CephKP |
| Raouf 2020 | Egypt | LMI | EMR | Mixed | Hospital | CRKP,  CREC |
| Rath 2012 | India | LMI | SEAR | Not reported | Hospital | CephEC,  CephEnt |
| Rath 2015 | India | LMI | SEAR | Mixed | Hospital | CREC,  CephEC |
| Raza 2019 | India | LMI | SEAR | Mixed | Hospital | CRAB,  CREC,  CephEC |
| Rizvi 2013 | India | LMI | SEAR | Mixed | Hospital | MRSA |
| Sahu 2016 | India | LMI | SEAR | Mixed | Cardiac surgical ICU | CREC,  CephEC,  CREnt,  CephEnt,  VSA |
| Salem-Bekhit 2014 | Egypt | LMI | EMR | Mixed | Hospital | MRSA,  VSA,  VMRSA |
| Essawy 2018 | Egypt | LMI | EMR | Mixed | Departments of medical microbiology, immunology and urology (non-ICU) | CREC,  CephEC |
| Hamam 2018 | Egypt | LMI | EMR | Mixed | ICU | CRAB |
| Sanou 2015 | Burkina Faso | LI | AFR | Mixed | Urology unit (non-ICU) | CREC,  CRKP |
| Saravu 2015 | India | LMI | SEAR | Mixed | ICU | MRSA,  CRAB,  CRPA,  CRKP |
| Sarkar 2016 | India | LMI | SEAR | Mixed | ICU | CRKP,  CephKP |
| Sateesh 2017 | India | LMI | SEAR | Mixed | ICU | CRPA |
| See 2013 | Egypt | LMI | EMR | Mixed | ICU | MRSA,  CREC,  CRKP |
| Seni 2013 | Uganda | LI | AFR | Mixed | Hospital | MRSA,  VSA |
| Shah 2019 | Pakistan | LMI | EMR | Mixed | ICU | CRAB,  CRKP,  VMRSA |
| Shalini 2010 | India | LMI | SEAR | Mixed | ICU | MRSA,  VSA,  VMRSA,  CRKP,  CephKP,  CRKP |
| Sharaf 2016 | Egypt | LMI | EMR | Mixed | Emergency and surgical ICU | CRAB |
| Sharan 2016 | India | LMI | SEAR | Mixed | Hospital | CRPA,  CRAB |
| Sharan 2013 | India | LMI | SEAR | Mixed | Non-ICU | MRSA,  VSA,  VMRSA,  CREC,  CRKP,  CRPA,  CephKP,  CephEC |
| Sharma 2013 | India | LMI | SEAR | Mixed | Hospital | MRSA,  VSA,  VMRSA |
| Shimi 2015 | Morocco | LMI | EMR | Mixed | ICU | CRPA,  CRAB,  MRSA |
| Shrestha 2019a | Nepal | LMI | SEAR | Mixed | Hospital | MRSA |
| Shrestha 2019b | Nepal | LMI | SEAR | Mixed | Hospital | MRSA,  VMRSA,  CREC,  CephEC,  CRKP,  CephKP |
| Sohail 2017 | Pakistan | LMI | EMR | Mixed | Hospital | MRSA,  VMRSA |
| Syed 2013 | Pakistan | LMI | EMR | Mixed | Hospital | CRKP,  CREC,  CRPA |
| Taha 2019 | Egypt | LMI | EMR | Mixed | Hospital | MRSA,  VMRSA |
| Talaat 2016 | Egypt | LMI | EMR | Mixed | ICU | CREC,  MRSA |
| Tambuwal 2020 | Nigeria | LMI | AFR | Mixed | Hospital | MRSA |
| Tolera 2018 | Ethiopia | LI | AFR | Mixed | Hospital | MRSA |
| Tran 2017 | Vietnam | LMI | WPR | Adults | ICU | CRPA,  CRAB |
| Trifi 2017 | Tunisia | LMI | EMR | Mixed | ICU | CRAB,  CRPA,  MRSA |
| Ul Haq 2018 | Pakistan | LMI | EMR | Adults | ICU | CRPA,  VMRSA,  VSA,  MRSA,  CREC |
| van der Meeren 2014 | Mozambique | LI | AFR | Mixed | Surgical Department (non-ICU) | MRSA,  VSA |
| Vasanthi 2012 | India | LMI | SEAR | Mixed | Hospital | MRSA |
| Le Minh 2015 | Vietnam | LMI | WPR | Mixed | ICU | CRAB |
| Vijaya 2014 | India | LMI | SEAR | Mixed | ICU | MRSA,  VSA,  VMRSA,  CephEC,  CREC |
| Vijayanarayana 2014 | India | LMI | SEAR | Adults | Hospital | MRSA,  CephKP,  CRKP,  CRPA,  CephEC,  CREC |
| Vijaykumar 2016 | India | LMI | SEAR | Adults | Medical ICU | CRKP,  CephKP,  CRPA |
| Vipin 2019 | India | LMI | SEAR | Mixed | Hospital | CRPA |
| Phu 2017 | Vietnam | LMI | WPR | Adults | ICU | MRSA,  CRAB,  CRKP,  CRPA |
| Phu 2016 | Vietnam | LMI | WPR | Adults | ICU | CRAB, CRPA,  CRKP,  MRSA |
| Dat 2017 | Vietnam | LMI | WPR | Adults | Hospital | CRKP,  CephKP |
| Yazigi 2019 | Syria | LI | EMR | Mixed | Hospital | CREC,  CephEC |

Table 2. Risk of Bias Assessment

| **Study** | **National / regional representativeness** | **Methodology of sample selection** | **Methodology for microbiological identification and antimicrobial susceptibility testing** | **Total risk of bias** |
| --- | --- | --- | --- | --- |
| Abdallah 2014 | High | High | High | High |
| Abd-Elmonsef 2018 | High | High | Low | High |
| Abdoulaye 2018 | High | High | High | High |
| Abdulall 2018 | High | Low | Low | Moderate |
| Afroz 2017 | High | Low | High | High |
| Agmy 2013 | High | High | Low | High |
| Ahmad 2014 | High | Low | High | High |
| Ahmad 2018 | High | High | High | High |
| Ahmed 2014 | High | High | Low | High |
| Fakhr 2018 | High | High | Low | High |
| Ahoyo 2014 | Low | Low | Low | Low |
| Alemayehu 2019 | High | Low | Low | Moderate |
| Alioua 2014 | High | Low | Low | Moderate |
| Amutha 2015 | High | High | Low | High |
| Anago 2015 | High | High | Low | High |
| Andrianarivelo 2017 | High | Low | Low | Moderate |
| Anuradha 2014 | High | Low | Low | Moderate |
| Arya 2014 | High | Low | Low | Moderate |
| Ateba 2013 | High | High | Low | High |
| Awoke 2019 | High | Low | Low | Moderate |
| Azzab 2016 | High | Low | Low | Moderate |
| Babu 2011a | High | Low | Low | Moderate |
| Babu 2011b | High | Low | Low | Moderate |
| Bammigatti 2017 | High | Low | Low | Moderate |
| Bashir 2019 | High | Low | Low | Moderate |
| Bhadade 2017 | High | High | High | High |
| Bhatia 2017 | High | Low | Low | Moderate |
| Bhattacharya 2016 | High | Low | Low | Moderate |
| Biedenbach 2016 | High | Low | Low | Moderate |
| Bijapur 2015 | High | Low | Low | Moderate |
| Bishwas 2020 | High | Low | Low | Moderate |
| Chaari 2013 | High | Low | High | High |
| Chandrasekaran 2016 | High | Low | High | High |
| Chaudhury 2016 | High | Low | Low | Moderate |
| Chavan 2017 | High | Low | High | High |
| Rosenthal 2020 | High | Low | High | High |
| Datta 2014 | High | Low | Low | Moderate |
| Dayyab 2018 | High | High | Low | High |
| Doddamani 2013 | High | High | Low | High |
| Dubey 2013a | High | Low | Low | Moderate |
| El-Kholy 2012 | High | Low | Low | Moderate |
| El-Mahdy 2019 | High | Low | Low | Moderate |
| El-Mahdy 2018 | High | Low | Low | Moderate |
| El Mekes 2020 | High | Low | Low | Moderate |
| El-Nawawy 2019 | High | Low | Low | Moderate |
| El-Sahrigy 2019 | High | Low | Low | Moderate |
| Elmouaden 2019 | High | Low | Low | Moderate |
| Eman 2018 | High | Low | Low | Moderate |
| Hammad 2011 | High | Low | Low | Moderate |
| Fatima 2013 | High | Low | High | High |
| Feleke 2018 | High | Low | Low | Moderate |
| Ferjani 2015 | High | High | Low | High |
| Fouad 2013 | High | Low | Low | Moderate |
| Fox-Lewis 2018 | High | Low | High | High |
| Gaber 2020 | High | Low | Low | Moderate |
| Gashaw 2018 | High | Low | High | High |
| Golia 2013 | High | Low | Low | Moderate |
| Gupta 2018 | High | Low | Low | Moderate |
| Gupta 2017 | High | Low | Low | Moderate |
| Hashem 2017 | High | Low | Low | Moderate |
| Hassan 2017 | High | Low | Low | Moderate |
| Hassuna 2020 | High | Low | Low | Moderate |
| Hassuna 2016 | High | Low | Low | Moderate |
| Hope 2019 | High | Low | Low | Moderate |
| Hossain 2014 | High | High | Low | High |
| Iliyasu 2020 | High | Low | High | High |
| Iliyasu 2016 | High | Low | High | High |
| Iliyasu 2018 | High | Low | Low | Moderate |
| Jadhav 2020 | High | High | High | High |
| Jain 2017 | High | High | Low | High |
| Jyoti 2016 | High | Low | Low | Moderate |
| Kahsay 2014 | High | Low | Low | Moderate |
| Kalayu 2019 | High | Low | Low | Moderate |
| Khairy 2020 | High | High | Low | High |
| Khalid 2018 | High | Low | High | High |
| Khan 2017 | High | Low | High | High |
| Khatun 2015 | High | High | Low | High |
| Khurana 2017 | High | Low | Low | Moderate |
| Kishk 2020 | High | Low | Low | Moderate |
| Kotb 2020 | High | Low | High | High |
| Kumar 2018a | High | Low | Low | Moderate |
| Labi 2019 | Low | High | High | High |
| Labib 2018 | High | Low | Low | Moderate |
| Lachhab 2017 | High | Low | Low | Moderate |
| Lakoh 2020 | High | Low | High | High |
| Le 2016 | High | Low | High | High |
| Mahfoud 2015 | High | Low | Low | Moderate |
| Esmat 2018 | High | Low | High | High |
| Soliman 2018 | High | Low | Low | Moderate |
| Manjhi 2018 | High | Low | High | High |
| Maoulainine 2014 | High | Low | High | High |
| Azab 2015 | High | High | High | High |
| Mawalla 2011 | High | Low | Low | Moderate |
| Mehta 2014 | High | High | Low | High |
| Mitharwal 2016 | High | Low | High | High |
| Moemen 2015 | High | Low | Low | Moderate |
| Mohanty 2017 | High | Low | Low | Moderate |
| Moolchandani 2017 | High | Low | Low | Moderate |
| Motbainor 2020 | High | Low | Low | Moderate |
| Mundhada 2015 | High | High | Low | High |
| Murphy 2016 | High | Low | Low | Moderate |
| Narula 2020 | High | Low | High | High |
| Negi 2015 | High | Low | Low | Moderate |
| Niranjan 2011 | High | Low | High | High |
| Njoku 2019 | High | Low | High | High |
| Nwankwo 2014 | High | Low | Low | Moderate |
| Olajubu 2012 | High | Low | High | High |
| Padmavathy 2018 | High | Low | Low | Moderate |
| Padmini 2019 | High | High | Low | High |
| Pal 2019 | High | Low | Low | Moderate |
| Parajuli 2017 | High | Low | Low | Moderate |
| Pathak 2014 | High | Low | Low | Moderate |
| Patil 2015 | High | Low | Low | Moderate |
| Peters 2019 | High | Low | Low | Moderate |
| Prakash 2013 | High | High | Low | High |
| Prakash 2017 | High | Low | Low | Moderate |
| Rafai 2015 | High | Low | High | High |
| Rafiq 2019 | High | Low | High | High |
| Ramakrishnan 2019 | High | Low | High | High |
| Raouf 2020 | High | Low | Low | Moderate |
| Rath 2012 | High | High | Low | High |
| Rath 2015 | High | Low | High | High |
| Raza 2019 | High | High | Low | High |
| Rizvi 2013 | High | Low | Low | Moderate |
| Sahu 2016 | High | Low | High | High |
| Salem-Bekhit 2014 | High | Low | Low | Moderate |
| Essawy 2018 | High | High | Low | High |
| Hamam 2018 | High | Low | Low | Moderate |
| Sanou 2015 | High | Low | Low | Moderate |
| Saravu 2015 | High | Low | Low | Moderate |
| Sarkar 2016 | High | Low | Low | Moderate |
| Sateesh 2017 | High | Low | High | High |
| See 2013 | High | Low | High | High |
| Seni 2013 | High | Low | Low | Moderate |
| Shah 2019 | High | Low | High | High |
| Shalini 2010 | High | Low | Low | Moderate |
| Sharaf 2016 | High | Low | Low | Moderate |
| Sharan 2016 | High | High | Low | High |
| Sharan 2013 | High | Low | High | High |
| Sharma 2013 | High | Low | Low | Moderate |
| Shimi 2015 | High | Low | High | High |
| Shrestha 2019a | High | Low | Low | Moderate |
| Shrestha 2019b | High | Low | Low | Moderate |
| Sohail 2017 | High | Low | Low | Moderate |
| Syed 2013 | High | High | High | High |
| Taha 2019 | High | Low | Low | Moderate |
| Talaat 2016 | High | Low | High | High |
| Tambuwal 2020 | High | Low | Low | Moderate |
| Tolera 2018 | High | Low | Low | Moderate |
| Tran 2017 | High | Low | Low | Moderate |
| Trifi 2017 | High | Low | High | High |
| Ul Haq 2018 | High | Low | High | High |
| van der Meeren 2014 | High | Low | High | High |
| Vasanthi 2012 | High | Low | High | High |
| Le Minh 2015 | High | Low | Low | Moderate |
| Vijaya 2014 | High | Low | High | High |
| Vijayanarayana 2014 | High | High | High | High |
| Vijaykumar 2016 | High | Low | Low | Moderate |
| Vipin 2019 | High | High | Low | High |
| Phu 2017 | High | Low | Low | Moderate |
| Phu 2016 | High | Low | High | High |
| Dat 2017 | High | Low | Low | Moderate |
| Yazigi 2019 | High | High | High | High |

The risk of bias (RoB) was judged for three domains: (i) national or regional representativeness of included patients/HAI isolates, (ii) sample selection method, and (iii) use of a sound microbiological method for pathogen identification and AST. The RoB for national or regional representativeness was judged as “low” if the study explicitly used an appropriate method to ensure the representativeness of the included patients/HAI isolates. The RoB for sample selection was judged as “low” if the study included all patients/HAI isolates in the study period (e.g. by consecutive inclusion of all patients/isolates) or used some form of random selection. If the study used established methods for pathogen identification and AST (e.g. automated systems, such as Vitek, Phoenix, or BacTec, as well as AST guidelines, such as CLSI and EUCAST), the RoB for the microbiological method was judged as “low”. Otherwise, the RoB was adjudged as high for the three criteria. The total RoB was judged as “low “ if the RoB was low in all three domains. The total RoB was judged as “moderate” if the RoB was “low” in two domains. The total RoB was judged as “high” if the the RoB was “high” in two or more domains.

References

1. A A, H G, H O. Early ICU energy deficit: Is it a risk factor for ventilator-associated pneumonia? Vol. 63, 2014:3-4.

2. Abd-Elmonsef MME, Elsharawy D, Abd-Elsalam AS. Mechanical ventilator as a major cause of infection and drug resistance in intensive care unit. Environmental Science and Pollution Research 2018; 25(31): 30787-92.

3. Abdoulaye O, Amadou MLH, Amadou O, et al. Epidemiological and bacteriological features of surgical site infections (ISO) in the Division of Surgery at the Niamey National Hospital (HNN). Pan African Medical Journal 2018; 31.

4. Abdulall AK, Tawfick MM, El Manakhly AR, El Kholy A. Carbapenem-resistant Gram-negative bacteria associated with catheter-related bloodstream infections in three intensive care units in Egypt. European Journal of Clinical Microbiology & Infectious Diseases 2018; 37(9): 1647-52.

5. Afroz H, Fakruddin M, Masud MR, Islam K. Incidence of and risk factors for hospital acquired infection in a tertiary care hospital of Dhaka, Bangladesh. Bangladesh Journal of Medical Science 2017; 16(3): 358-69.

6. Agmy G, Mohamed S, Gad Y, Farghally E, Mohammedin H, Rashed H. Bacterial Profile, Antibiotic Sensitivity and Resistance of Lower Respiratory Tract Infections in Upper Egypt. Mediterranean Journal of Hematology and Infectious Diseases 2013; 5(1): 1-7.

7. Ahmad MK, Asrar A. Prevalence of Methicillin Resistant Staphylococcus aureus in pyogenic community and hospital acquired skin and soft tissues infections. Journal of the Pakistan Medical Association 2014; 64(8): 892-5.

8. Ahmad R. Evaluation of the gram-negative bacilli causing surgical-site infections and their sensitivity to antibiotics in al-mowasat hospital, Damascus, Syria. Research Journal of Pharmacy and Technology 2018; 11(5): 2070-3.

9. Ahmed EF, Gad GFM, Abdalla AM, Hasaneen AM, Abdelwahab SF. Prevalence of Methicillin Resistant Staphylococcus aureus among Egyptian Patients after Surgical Interventions. Surgical Infections 2014; 15(4): 404-11.

10. Ahmed F, Fayza F. Bacterial pattern and risk factors of hospital acquired infections in a tertiary care hospital, Egypt. Vol. 27, 2018:9-16.

11. Ahoyo TA, Bankole HS, Adeoti FM, et al. Prevalence of nosocomial infections and anti-infective therapy in Benin: results of the first nationwide survey in 2012. Antimicrobial Resistance and Infection Control 2014; 3.

12. Alemayehu T, Tadesse E, Ayalew S, et al. High burden of nosocomial infections caused by multi-drug resistant pathogens in pediatric patients at hawassa university comprehensive specialized hospital. Ethiopian Medical Journal 2019.

13. Alioua MA, Labid A, Amoura K, Bertine M, Gacemi-Kirane D, Dekhil M. Emergence of the European ST80 clone of community-associated methicillin-resistant Staphylococcus aureus as a cause of healthcare-associated infections in Eastern Algeria. Medecine et Maladies Infectieuses 2014; 44(4): 180-3.

14. Amutha B, Viswanathan T. Incidences of staphylococcus species in surgical site infections-prospective study from tertiary care center, Coimbatore, Tamilnadu. International Journal of Pharmacy and Pharmaceutical Sciences 2015; 7(5): 225-8.

15. Anago E, Ayi-Fanou L, Akpovi CD, et al. Antibiotic resistance and genotype of beta-lactamase producing Escherichia coli in nosocomial infections in Cotonou, Benin. Annals of Clinical Microbiology and Antimicrobials 2015; 14.

16. Andrianarivelo AM, Andriamandimbisoa TH, Rakotondraoelina LM, et al. Status of resistance to antimicrobial agents of staphylococcus aureus strains at the laboratory of microbiology of the hu-jra antananarivo. African Journal of Clinical and Experimental Microbiology 2017; 18(3): 133-8.

17. Anuradha M, Dandekar RH. Antibiotic sensitivity pattern among hospital acquired infections in a tertiary care hospital: An attempt for formulating antibiotic policy. International Journal of Pharma and Bio Sciences 2014; 5(2): B902-B13.

18. Arya SC, Agarwal N, Agarwal S, Singh K, Wadhwa D. Impact of clinical syndrome-wise categorisation of antimicrobial susceptibility profiles on nosocomial isolates in a tertiary care hospital in Delhi, India. Journal of Global Antimicrobial Resistance 2014; 2(1): 23-6.

19. Ateba NS, Ngaba GP, Ebongue CO, et al. Susceptibility to colistin of multi-resistant pseudomonas aeruginosa isolated in Douala Laquintinie Hospital, Cameroon. African Journal of Pathology and Microbiology 2013.

20. Awoke N, Kassa T, Teshager L. Magnitude of Biofilm Formation and Antimicrobial Resistance Pattern of Bacteria Isolated from Urinary Catheterized Inpatients of Jimma University Medical Center, Southwest Ethiopia. International journal of microbiology 2019; 2019.

21. Azzab MM, El-Sokkary RH, Tawfeek MM, Gebriel MG. Multidrug-resistant bacteria among patients with ventilator-associated pneumonia in an emergency intensive care unit, Egypt. Eastern Mediterranean Health Journal 2016; 22(12): 894-903.

22. Babu KVY, Jayasimha VL, Basavarajappa KG, et al. A comparative study of ventilator-associated pneumonia and ventilator associated tracheobronchitis: Incidence, outcome, risk factors. Biosciences Biotechnology Research Asia 2011; 8(1): 195-203.

23. Babu KVY, Niranjan HP, Vijayanath V, Anitha MR, Raju GM. The Influence of Metallo-Beta-Lactamase Production and Predisposing Risk Factors on Mortality in Pseudomonas aeruginosa Nosocomial Infections. Journal of Pure and Applied Microbiology 2011; 5(2): 761-8.

24. Bammigatti C, Doradla S, Belgode HN, Kumar H, Swaminathan RP. Healthcare Associated Infections in a Resource Limited Setting. Journal of Clinical and Diagnostic Research 2017; 11(1): OC1-OC4.

25. Bashir A, Garba I, Aliero AA, et al. Superbugs-related prolonged admissions in three tertiary hospitals, Kano State, Nigeria. Pan African Medical Journal 2019; 32.

26. Bhadade R, Harde M, desouza R, More A, Bharmal R. Emerging Trends of Nosocomial Pneumonia in Intensive Care Unit of a Tertiary Care Public Teaching Hospital in Western India. Annals of African medicine 2017; 16(3): 107-13.

27. Bhatia M, Loomba PS, Mishra B, Dogra V. Comparative Evaluation of In-Vitro Doripenem Susceptibility with Other Carbapenem Antibiotics among Gram Negative Bacterial Isolates Obtained from VAP Patients in a Super-Speciality Hospital: A Pilot Study. International Journal of Medical Research & Health Sciences 2017; 6(4): 36-41.

28. Bhattacharya S, Pal K, Jain S, Chatterjee SS, Konar J. Surgical site infection by methicillin resistant staphylococcus aureus– On decline? Journal of Clinical and Diagnostic Research 2016; 10(9): DC32-DC6.

29. Biedenbach DJ, Phan Trong G, Pham Hung V, et al. Antimicrobial-resistant Pseudomonas aeruginosa and Acinetobacter baumannii From Patients With Hospital-acquired or Ventilator-associated Pneumonia in Vietnam. Clinical Therapeutics 2016; 38(9): 2098-105.

30. Bijapur GAM, Maulingkar SV, Greeshma B, Usman SM. Multidrug resistant Escherichia coli in nosocomial urinary tract infections at a tertiary care hospital in Kerala, India. Open Infectious Diseases Journal 2015; 9(1): 30-4.

31. Bishwas A, Hemavathi, Shenoy P. Microbial Profile of Ventilator Associated Pneumonia in a Medical Intensive Care Unit of a Tertiary Care Hospital in Bangalore. Journal of Evolution of Medical and Dental Sciences-Jemds 2020; 9(19): 1539-43.

32. Chaari A, Mnif B, Bahloul M, et al. Acinetobacter baumannii ventilator-associated pneumonia: epidemiology, clinical characteristics, and prognosis factors. International Journal of Infectious Diseases 2013; 17(12): E1225-E8.

33. Chandrasekaran K, Saeed K, Gandhiraj D, Mohanta GP, Rajasekaran A. Study on adherence to prophylactic antimicrobials use guidelines in surgical wards of an Indian private corporate hospital. International Journal of Pharmaceutical Research and Allied Sciences 2016; 5(2): 280-92.

34. Chaudhury A, Rani AS, Kalawat U, Sumant S, Verma A, Venkataramana B. Antibiotic resistance & pathogen profile in ventilator-associated pneumonia in a tertiary care hospital in India. Indian Journal of Medical Research 2016; 144: 440-6.

35. Chavan AR, Kelkar V. Study of healthcare-associated infections in surgical unit in a newly established tertiary care hospital of Nanded, Maharashtra, India. International Journal of Surgery Open 2017; 9: 30-5.

36. Daniel Rosenthal V, Gupta D, Rajhans P, et al. Six-year multicenter study on short-term peripheral venous catheters-related bloodstream infection rates in 204 intensive care units of 57 hospitals in 19 cities of India: International Nosocomial Infection Control Consortium (INICC) findings. American Journal of Infection Control 2020; 48(9): 1001-8.

37. Datta P, Rani H, Chauhan R, Gombar S, Chander J. Health-care-associated infections: Risk factors and epidemiology from an intensive care unit in Northern India. Indian Journal of Anaesthesia 2014; 58(1): 30-5.

38. Dayyab FM, Iliyasu G, Aminu A, et al. A prospective study of hospital-acquired infections among adults in a tertiary hospital in north-western Nigeria. Transactions of the Royal Society of Tropical Medicine and Hygiene 2018; 112(1): 36-42.

39. Doddamani PK, Neelima. Bacteriological profile of surgical site infection in rural hospital in R.R district. International Journal of Pharma and Bio Sciences 2013; 4(3): B217-B21.

40. Dubey D, Rath S, Sahu MC, Pattnaik L, Debata NK, Padhy RN. Surveillance of infection status of drug resistant Staphylococcus aureus in an Indian teaching hospital. Asian Pacific Journal of Tropical Disease 2013; 3(2): 133-42.

41. El Mekes A, Zahlane K, Said LA, Ouafi AT, Barakate M. The clinical and epidemiological risk factors of infections due to multi-drug resistant bacteria in an adult intensive care unit of University Hospital Center in Marrakesh-Morocco. Journal of Infection and Public Health 2020; 13(4): 637-43.

42. El-Kholy A, Saied T, Gaber M, et al. Device-associated nosocomial infection rates in intensive care units at Cairo University hospitals: First step toward initiating surveillance programs in a resource-limited country. American Journal of Infection Control 2012; 40(6): E216-E20.

43. El-Mahdy R, El-Kannishy G. Virulence factors of carbapenem-resistant pseudomonas aeruginosa in hospital-acquired infections in Mansoura, Egypt. Infection and Drug Resistance 2019; 12((El-Mahdy R., rashaamr@mans.edu.eg) Department of Medical Microbiology And Immunology, Faculty of Medicine, Mansoura University, Mansoura, Egypt(El-Kannishy G.) Department of Internal Medicine, Faculty of Medicine, Mansoura University, Mansoura, Egypt): 3455-61.

44. El-Mahdy R, El-Kannishy G, Salama H. Hypervirulent Klebsiella pneumoniae as a hospital-acquired pathogen in the intensive care unit in Mansoura, Egypt. Germs 2018; 8(3): 140-6.

45. Elmouaden C, Laglaoui A, Ennanei L, Bakkali M, Abid M. Virulence genes and antibiotic resistance of Pseudomonas aeruginosa isolated from patients in the Northwestern of Morocco. Journal of Infection in Developing Countries 2019; 13(10): 892-8.

46. El-Nawawy A, Ramadan MAF, Antonios MAM, Arafa SAF, Hamza E. Bacteriologic profile and susceptibility pattern of mechanically ventilated paediatric patients with pneumonia. Journal of Global Antimicrobial Resistance 2019; 18((El-Nawawy A.; Ramadan M.A.-F.; Antonios M.A.-M., malakmanal@yahoo.com; Hamza E.) Department of Pediatrics, Alexandria University, Faculty of Medicine, El-Shatby Hospital Alexandria, Egypt(Arafa S.A.-F.) Medical Microbiology and Immunology, Alexandria Uni): 88-94.

47. El-Sahrigy SAF, Shouman MG, Ibrahim HM, et al. Prevalence and Anti-Microbial Susceptibility of Hospital Acquired Infections in Two Pediatric Intensive Care Units in Egypt. Open access Macedonian journal of medical sciences 2019; 7(11): 1744-9.

48. Eman EM, Hesham EM. Characterization of carbapenem-resistant Acinetobacter baumannii isolated from intensive care unit, Egypt. Vol. 27, 2018:85-91.

49. Enas H, Mohamed S, Hassan S. Neurology intensive care unit-symptomatic nosocomial urinary tract infections: management and prognosis. Vol. 20, 2011:117-24.

50. Fatima SS, Gillani S, Naib J, Sharafat Z, Mazhar T, Naveed P. Anti-microbial sensitivity patterns of bacterial isolates from surgical site infections in obstetrics and gynaecology. Journal of Medical Sciences (Peshawar) 2013; 21(4): 201-5.

51. Feleke T, Eshetie S, Dagnew M, et al. Multidrug-resistant bacterial isolates from patients suspected of nosocomial infections at the University of Gondar Comprehensive Specialized Hospital, Northwest Ethiopia. BMC research notes 2018; 11(1): 602-.

52. Ferjani S, Saidani M, Amine FS, Ben Boubaker IB. A comparative study of antimicrobial resistance rates and phylogenetic groups of community-acquired versus hospital-acquired invasive Escherichia coli. Medecine Et Maladies Infectieuses 2015; 45(4): 133-8.

53. Fouad M, Attia AS, Tawakkol WM, Hashem AM. Emergence of carbapenem-resistant Acinetobacter baumannii harboring the OXA-23 carbapenemase in intensive care units of Egyptian hospitals. International Journal of Infectious Diseases 2013; 17(12): e1252-e4.

54. Fox-Lewis A, Takata J, Miliya T, et al. Antimicrobial Resistance in Invasive Bacterial Infections in Hospitalized Children, Cambodia, 2007-2016. Emerging Infectious Diseases 2018; 24(5): 841-51.

55. Gaber SN, Hemeda EEM, Elsayeh HAS, Abdel Wahed WY, Khalil MAF, Ibrahim EG. Propolis extract: A possible antiseptic oral care against multidrug-resistant non-fermenting bacteria isolated from non-ventilator hospital-acquired pneumonia. Journal of Pure and Applied Microbiology 2020; 14(1): 123-31.

56. Gashaw M, Berhane M, Bekele S, et al. Emergence of high drug resistant bacterial isolates from patients with health care associated infections at Jimma University medical center: a cross sectional study. Antimicrobial Resistance and Infection Control 2018; 7.

57. Golia S, Sangeetha KT, Vasudha CL. Microbial profile of early and late onset ventilator associated pneumonia in the intensive care unit of a tertiary care hospital in Bangalore, India. Journal of Clinical and Diagnostic Research 2013; 7(11): 2462-6.

58. Gupta N, Soneja M, Ray Y, et al. Nosocomial pneumonia: Search for an empiric and effective antibiotic regimen in high burden tertiary care centre. Drug Discoveries and Therapeutics 2018; 12(2): 97-100.

59. Gupta R, Malik A, Rizvi M, Ahmed M, Singh A. Epidemiology of multidrug-resistant Gram-negative pathogens isolated from ventilator-associated pneumonia in ICU patients. Journal of Global Antimicrobial Resistance 2017; 9((Gupta R., sunita1457@gmail.com; Malik A.; Rizvi M.; Singh A.) Department of Microbiology, Jawaharlal Nehru Medical College (JNMC), Aligarh Muslim University (AMU), Aligarh, Uttar Pradesh, India(Ahmed M.) Department of Anaesthesiology, JNMC, AMU, Aligarh): 47-50.

60. Hashem AA, El Fadeal NMA, Shehata AS. In vitro activities of vancomycin and linezolid against biofilm-producing methicillin-resistant staphylococci species isolated from catheter-related bloodstream infections from an Egyptian tertiary hospital. Journal of Medical Microbiology 2017; 66(6): 744-52.

61. Hassan RH, Eldegla H, Elmorsy F, Eldars WM. Clinical and microbiological characteristics of healthcare-associated infections in a tertiary care pediatric hospital. Egyptian Pediatric Association Gazette 2017; 65(4): 127-31.

62. Hassuna NA. Molecular Detection of the Virulent ExoU Genotype of Pseudomonas aeruginosa Isolated from Infected Surgical Incisions. Surgical Infections 2016; 17(5): 610-4.

63. Hassuna NA, AbdelAziz RA, Zakaria A, Abdelhakeem M. Extensively-Drug Resistant Klebsiella pneumoniae Recovered From Neonatal Sepsis Cases From a Major NICU in Egypt. Frontiers in Microbiology 2020; 11((Hassuna N.A., nohaanwar@mu.edu.eg) Department of Medical Microbiology and Immunology, Faculty of Medicine, Minia University, Minia, Egypt(AbdelAziz R.A.) Department of Pediatrics, Faculty of Medicine, Minia University, Minia, Egypt(Zakaria A.) Biotechnol).

64. Hope D, Ampaire L, Oyet C, Muwanguzi E, Twizerimana H, Apecu RO. Antimicrobial resistance in pathogenic aerobic bacteria causing surgical site infections in Mbarara regional referral hospital, Southwestern Uganda. Scientific Reports 2019; 9.

65. Hossain MD, Ahsan S, Kabir MS. Antibiotic resistance patterns of uropathogens isolated from catheterized and noncatheterized patients in Dhaka, Bangladesh. Tzu Chi Medical Journal 2014; 26(3): 127-31.

66. Iliyasu G, Daiyab FM, Tiamiyu AB, et al. Nosocomial infections and resistance pattern of common bacterial isolates in an intensive care unit of a tertiary hospital in Nigeria: A 4-year review. Journal of Critical Care 2016; 34: 116-20.

67. Iliyasu G, Dayyab FM, Abubakar S, et al. Laboratory-confirmed hospital-acquired infections: An analysis of a hospital's surveillance data in Nigeria. Heliyon 2018; 4(8).

68. Iliyasu G, Dayyab FM, Aminu A, et al. Epidemiology and Microbiology of Bacterial Bloodstream Infection in a Tertiary Hospital in Nigeria. Infectious Diseases in Clinical Practice 2020; 28(1): 16-21.

69. Jadhav NN, Deokar V. Bacterial and fungal isolates from endotracheal tube secretions culture and their antibiogram in patients with ventilator associated pneumonia. International Journal of Research in Pharmaceutical Sciences 2020; 11(3): 4923-9.

70. Jain S, Gopi A, Samreen F, Madhulatha CK. METHICILLIN-RESISTANT STAPHYLOCOCCUS AUREUS, EXTENDED SPECTRUM BETALACTAMASE AND METALLOBETALACTAMASE PRODUCTION AMONG ORGANISMS CAUSING SURGICAL SITE INFECTIONS AT A TERTIARY CARE HOSPITAL IN BANGALORE. Journal of Evolution of Medical and Dental Sciences-Jemds 2017; 6(72): 5123-7.

71. Jyoti K, Shalini M S, Shrikala B, M C, Gopalkrishna K B. Healthcare-associated methicillin-resistant Staphylococcus Aureus: clinical characteristics and antibiotic resistance profile with emphasis on macrolide-lincosamide-streptogramin B resistance. Vol. 16, 2016:175-81.

72. Kahsay A, Mihret A, Abebe T, Andualem T. Isolation and antimicrobial susceptibility pattern of Staphylococcus aureus in patients with surgical site infection at Debre Markos Referral Hospital, Amhara Region, Ethiopia. Archives of public health = Archives belges de sante publique 2014; 72(1): 16-.

73. Kalayu AA, Diriba K, Girma C, Abdella E. Incidence and bacterial etiologies of surgical site infections in a Public Hospital, Addis Ababa, Ethiopia. Open Microbiology Journal 2019; 13(1): 301-7.

74. Khairy RMM, Mahmoud MS, Shady RR, Esmail MAM. Multidrug-resistant Klebsiella pneumoniae in hospital-acquired infections: Concomitant analysis of antimicrobial resistant strains. International Journal of Clinical Practice 2020; 74(4).

75. Khalid H, Nafees F, Khaliq MA. Infective organisms and their changing antibiotic sensitivity trends in surgical site infection after orthopedic implant surgeries. Pakistan Journal of Medical and Health Sciences 2018; 12(3): 1256-8.

76. Khan ID, Basu A, Kiran S, Trivedi S, Pandit P, Chattoraj A. Device-Associated Healthcare-Associated Infections (DA-HAI) and the caveat of multiresistance in a multidisciplinary intensive care unit. Medical journal, Armed Forces India 2017; 73(3): 222-31.

77. Khatun MN, Farzana R, Lopes BS, Shamsuzzaman SM. Molecular characterization and resistance profile of nosocomial Acinetobacter baumannii intensive care unit of tertiary care hospital in Bangladesh. Bangladesh Medical Research Council bulletin 2015; 41(2): 101-7.

78. Khurana S, Mathur P, Kumar S, et al. Incidence of Ventilator-associated Pneumonia and Impact of Multidrug-Resistant Infections on Patient's Outcome: Experience at an Apex Trauma Centre in North India. Indian Journal of Medical Microbiology 2017; 35(4): 504-10.

79. Kishk RM, Abdalla MO, Hashish AA, et al. Efflux MexAB-Mediated Resistance in P. aeruginosa Isolated from Patients with Healthcare Associated Infections. Pathogens 2020; 9(6).

80. Kotb S, Lyman M, Ismail G, et al. Epidemiology of Carbapenem-resistant Enterobacteriaceae in Egyptian intensive care units using National Healthcare-associated Infections Surveillance Data, 2011-2017. Antimicrobial Resistance and Infection Control 2020; 9(1).

81. Kumar S, Jan RA, Fomda BA, et al. Healthcare-Associated Pneumonia and Hospital-Acquired Pneumonia: Bacterial Aetiology, Antibiotic Resistance and Treatment Outcomes: A Study From North India. Lung 2018; 196(4): 469-79.

82. Labi AK, Obeng-Nkrumah N, Owusu E, et al. Multi-centre point-prevalence survey of hospital-acquired infections in Ghana. Journal of Hospital Infection 2019; 101(1): 60-8.

83. Labib JR, Ibrahim SK, Salem MR, Youssef MRL, Meligy B. Infection with gram-negative bacteria among children in a tertiary pediatric hospital in Egypt. American Journal of Infection Control 2018; 46(7): 798-801.

84. Lachhab Z, Frikh M, Maleb A, et al. Bacteraemia in Intensive Care Unit: Clinical, Bacteriological, and Prognostic Prospective Study. Canadian Journal of Infectious Diseases & Medical Microbiology 2017; 2017.

85. Lakoh S, Li L, Sevalie S, et al. Antibiotic resistance in patients with clinical features of healthcare-associated infections in an urban tertiary hospital in Sierra Leone: a cross-sectional study. Antimicrobial Resistance and Infection Control 2020; 9(1).

86. Le NK, Hf W, Vu PD, et al. High prevalence of hospital-acquired infections caused by gram-negative carbapenem resistant strains in Vietnamese pediatric ICUs. Medicine (United States) 2016; 95(27).

87. Mahfoud M, Al Najjar M, Hamzeh AR. Multidrug resistance in Pseudomonas aeruginosa isolated from nosocomial respiratory and urinary infections in Aleppo, Syria. Journal of Infection in Developing Countries 2015; 9(2): 210-3.

88. Mamdouh E, Asmaa G, Hala A, Alaa R. Surveillance of surgical site infection in General Surgery Department at Sohag University Hospital. Vol. 27, 2018:159-66.

89. Manar S, Sherif M. Prevalence of ISAba1-blaOXA-23, ISAba125-blaNDM-1, and armA genes in high-level aminoglycoside resistant Acinetobacter baumanni. Vol. 27, 2018:165-71.

90. Manjhi M, Das S, Pal M, Saha I, Reddy SA. Incidence, risk factors, clinico-microbiological profile, change in ventilator settings needed and outcome of 135 ventilator associated pneumonia cases in pediatric intensive care unit (PICU) of a tertiary care centre in Eastern India. Journal of Pediatric and Neonatal Individualized Medicine 2018; 7(1).

91. Maoulainine FMR, Elidrissi NS, Chkil G, et al. Epidemiology of nosocomial bacterial infection in neonatal intensive care unit in Morocco. Archives De Pediatrie 2014; 21(9): 938-43.

92. Marwa M A, Atef S, Mai M. OXA-10 and GES-1 extended-spectrum beta-lactamases play a major role in causing antibiotic resistance of Pseudomonas aeruginosa isolated from nosocomial infections in Ismailia, Egypt. Vol. 24, 2015:81-8.

93. Mawalla B, Mshana SE, Chalya PL, Imirzalioglu C, Mahalu W. Predictors of surgical site infections among patients undergoing major surgery at Bugando Medical Centre in Northwestern Tanzania. Bmc Surgery 2011; 11.

94. Mehta S, Sahni N, Singh VA, Bunger R, Garg T, Shinu P. Nosocomial wound infection amongst post operative patients and their antibiograms at tertiary care hospital in India. African Journal of Clinical and Experimental Microbiology 2014; 15(2): 60-8.

95. Mitharwal SM, Yaddanapudi S, Bhardwaj N, Gautam V, Biswal M, Yaddanapudi L. Intensive care unit-acquired infections in a tertiary care hospital: An epidemiologic survey and influence on patient outcomes. American Journal of Infection Control 2016; 44(7): e113-e7.

96. Moemen D, Tawfeek D, Badawy W. Healthcare-associated vancomycin resistant &lt;italic&gt;Enterococcus faecium&lt;/italic&gt; infections in the Mansoura University Hospitals intensive care units, Egypt. Braz j microbiol 2015; 46(3): 777-83.

97. Mohanty A, Shantikumar Singh T, Kabi A, Gupta P, Kumar P. Bacteriological profile and antibiotic sensitivity pattern of hospitalacquired septicemia in a tertiary care hospital in North East India. Asian Journal of Pharmaceutical and Clinical Research 2017; 10(11): 186-9.

98. Moolchandani K, Sastry AS, Deepashree R, Sistla S, Harish BN, Mandal J. Antimicrobial resistance surveillance among intensive care units of a tertiary care hospital in South India. Journal of Clinical and Diagnostic Research 2017; 11(2): DC01-DC7.

99. Motbainor H, Bereded F, Mulu W. Multi-drug resistance of blood stream, urinary tract and surgical site nosocomial infections of Acinetobacter baumannii and Pseudomonas aeruginosa among patients hospitalized at Felegehiwot referral hospital, Northwest Ethiopia: a cross-sectional study. Bmc Infectious Diseases 2020; 20(1).

100. Mundhada AS, Tenpe S. A study of organisms causing surgical site infections and their antimicrobial susceptibility in a tertiary care Government Hospital. Indian Journal of Pathology and Microbiology 2015; 58(2): 195-200.

101. Murphy RA, Okoli O, Essien I, et al. Multidrug-resistant surgical site infections in a humanitarian surgery project. Epidemiology and Infection 2016; 144(16): 3520-6.

102. Narula H, Chikara G, Gupta P. A prospective study on bacteriological profile and antibiogram of postoperative wound infections in a tertiary care hospital in Western Rajasthan. Journal of family medicine and primary care 2020; 9(4): 1927-34.

103. Negi V, Pal S, Juyal D, Sharma MK, Sharma N. Bacteriological Profile of Surgical Site Infections and Their Antibiogram: A Study From Resource Constrained Rural Setting of Uttarakhand State, India. Journal of clinical and diagnostic research : JCDR 2015; 9(10): DC17-20.

104. Niranjan HP, Jayasimha VL, Anitha MR, Vijayanath V, Basavarajappa KG. Study of Bacteriological Profile in Ventilator Associated Pneumonia. Journal of Pure and Applied Microbiology 2011; 5(2): 831-5.

105. Njoku CO, Njoku AN. Microbiological Pattern of Surgical Site Infection Following Caesarean Section at the University of Calabar Teaching Hospital. Open access Macedonian journal of medical sciences 2019; 7(9): 1430-5.

106. Nwankwo EO, Mofolorunsho CK, Akande AO. Aetiological agents of surgical site infection in a specialist hospital in kano, North-western Nigeria. Tanzania Journal of Health Research 2014; 16(4).

107. Olajubu AF, Osinupebi OA, Ismail L, Bosede O, Deji-Agboola AM. Pattern of hospital associated infections in a teaching hospital in Nigeria. Asian Pacific Journal of Tropical Disease 2012; 2(SUPPL2): S869-S73.

108. Padmavathy M, Priyanka S, Malini J. STAPHYLOCOCCUS AUREUS- HOSPITAL ACQUIRED AND COMMUNITY ACQUIRED, AND ANTIMICROBIAL TESTING IN A TERTIARY CARE HOSPITAL. Journal of Evolution of Medical and Dental Sciences-Jemds 2018; 7(40): 5186-90.

109. Padmini N, Kennedy Ajilda AA, Sivakumar N, Sureka I, Kumar RS, Selvakumar G. Genetic determination and characterization of extended spectrum β-lactamase producing Escherichia coli and Klebsiella pneumoniae in a tertiary care hospital, India. Indian Journal of Biotechnology 2019; 18(2): 145-50.

110. Pal S, Sayana A, Joshi A, Juyal D. Staphylococcus aureus: A predominant cause of surgical site infections in a rural healthcare setup of Uttarakhand. Journal of family medicine and primary care 2019; 8(11): 3600-6.

111. Parajuli NP, Acharya SP, Mishra SK, Parajuli K, Rijal BP, Pokhrel BM. High burden of antimicrobial resistance among gram negative bacteria causing healthcare associated infections in a critical care unit of Nepal. Antimicrobial Resistance and Infection Control 2017; 6.

112. Pathak A, Saliba EA, Sharma S, Mahadik VK, Shah H, Lundborg CS. Incidence and factors associated with surgical site infections in a teaching hospital in Ujjain, India. American Journal of Infection Control 2014; 42(1): E11-E5.

113. Patil HV, Mohite ST, Karande G, Patil VC, Patil SR, Pawar S. Incidence, bacteriology and risk factors of surgical site infection (SSI) at tertiary care teaching hospital. Research Journal of Pharmaceutical, Biological and Chemical Sciences 2015; 6(2): 1870-80.

114. Peters L, Olson L, Khu DTK, et al. Multiple antibiotic resistance as a risk factor for mortality and prolonged hospital stay: A cohort study among neonatal intensive care patients with hospital-acquired infections caused by gram-negative bacteria in Vietnam. Plos One 2019; 14(5).

115. Prakash D, Saxena RS. Prevalence and antimicrobial susceptibility pattern of Escherichia coli in hospital acquired and community acquired patients related to urinary tract infection in India. 2013.

116. Prakash PH, Rajan V, Gopal S. Predominance of SCCmec types IV and V among biofilm producing device-associated Staphylococcus aureus strains isolated from tertiary care hospitals in Mysuru, India. Enfermedades Infecciosas Y Microbiologia Clinica 2017; 35(4): 229-35.

117. Rafai C, Frank T, Manirakiza A, et al. Dissemination of IncF-type plasmids in multiresistant CTX-M-15-producing Enterobacteriaceae isolates from surgical-site infections in Bangui, Central African Republic. Bmc Microbiology 2015; 15.

118. Rafiq A, Ahsan Ul H, Hannan A, Choudhary AA. Prevalence of antibiotic resistant pathogens in post-orthopedic implant site. Medical Forum Monthly 2019; 30(3): 18-20.

119. Ramakrishnan K, Venugopal J, Easow JM, Ravishankar M. Incidence, Bacteriological Profile and Antibiotic Resistance Pattern of Catheter Associated Urinary Tract Infections in a Tertiary Care Hospital. Journal of Pure and Applied Microbiology 2019; 13(3): 1549-54.

120. Raouf M, Ghazal T, Kassem M, Agamya A, Amer A. Surveillance of surgical-site infections and antimicrobial resistance patterns in a tertiary hospital in Alexandria, Egypt. Journal of Infection in Developing Countries 2020; 14(3): 277-83.

121. Rath S, Padhy RN. Surveillance of multidrug resistance of 10 enteropathogens in a teaching hospital and in vitro efficacy of 25 ethnomedicinal plants used by an Indian aborigine. Asian Pacific Journal of Tropical Disease 2012; 2(SUPPL.1): S336-S46.

122. Rath S, Padhy RN. Prevalence of fluoroquinolone resistance in Escherichia coli in an Indian teaching hospital and adjoining communities. Journal of Taibah University Medical Sciences 2015; 10(4): 504-8.

123. Raza MS, Das BK, Goyal V, et al. Emerging multidrug resistance isolates of hospital-acquired bacterial meningitis in a tertiary care centre in North India. Journal of Medical Microbiology 2019; 68(11): 1585-90.

124. Rizvi M, Rizvi MW, Shaheen, et al. Emergence of coryneform bacteria as pathogens in nosocomial surgical site infections in a tertiary care hospital of North India. Journal of Infection and Public Health 2013; 6(4): 283-8.

125. Sahu M, Siddharth B, Choudhury A, et al. Incidence, microbiological profile of nosocomial infections, and their antibiotic resistance patterns in a high volume Cardiac Surgical Intensive Care Unit. Annals of Cardiac Anaesthesia 2016; 19(2): 281-7.

126. Salem-Bekhit MM. Phenotypic and Genotypic Characterization of Nosocomial Isolates of Staphylococcus aureus with Reference to Methicillin Resistance. Tropical Journal of Pharmaceutical Research 2014; 13(8): 1239-46.

127. Sally E, Mona R, Mina M, Mohamed G. Detection of extended spectrum beta-lactamase producing Escherichia coli among community-acquired and hospital-acquired urinary tract infections in Tanta University Hospital. Vol. 27, 2018:99-105.

128. Sanaa H, Samah A. Characterization of antimicrobial resistance and prevalence of OXA genes in the emerging threat Acinetobacter baumannii causing blood stream infection in ICU patients. Vol. 27, 2018:141-8.

129. Sanou I, Kabore A, Tapsoba E, Bicaba I, Ba A, Zango B. Nosocomial urinary infections at the urogoly unit of the national university hospital (yalgado Ouedraogo), Ouagadougou: Feb.-Sept. 2012. African Journal of Clinical and Experimental Microbiology 2015; 16(1): 1-6.

130. Saravu K, Prasad M, Eshwara VK, Mukhopadhyay C. Clinico-microbiological profile and outcomes of nosocomial sepsis in an Indian tertiary care hospital - a prospective cohort study. Pathogens and Global Health 2015; 109(5): 228-35.

131. Sarkar M, Raj HJ, Ghosh TK. Ventilator associated Pneumonia a challenge in intensive care unit acquired infection. Bangladesh Journal of Medical Science 2016; 15(4): 588-95.

132. Sateesh K, Anandam S, Pai V. BACTERIOLOGICAL PROFILE OF VENTILATOR ASSOCIATED PNEUMONIA IN A TERTIARY CARE CENTRE OF DAKSHINA KANNADA DIST. Journal of Evolution of Medical and Dental Sciences-Jemds 2017; 6(80): 5664-7.

133. See I, Lessa FC, ElAta OA, et al. Incidence and Pathogen Distribution of Healthcare-Associated Infections in Pilot Hospitals in Egypt. Infection Control and Hospital Epidemiology 2013; 34(12): 1281-8.

134. Seni J, Bwanga F, Najjuka CF, et al. Molecular Characterization of Staphylococcus aureus from Patients with Surgical Site Infections at Mulago Hospital in Kampala, Uganda. Plos One 2013; 8(6).

135. Shah AA, Jamil B, Naseem S, et al. Susceptibility pattern of tracheal tube isolates from Intensive Care Unit of Fauji Foundation Hospital Rawalpindi. Journal of the Pakistan Medical Association 2019; 69(7): 981-4.

136. Shalini S, Kranthi K, Gopalkrishna Bhat K. The microbiological profile of nosocomial infections in the intensive care unit. Journal of Clinical and Diagnostic Research 2010; 4(5): 3109-12.

137. Sharaf HE, Gerges MA. Nosocomial imipenem-resistant Acinetobacter Baumannii infections in intensive care units: Incidence and risk factors assessment. African Journal of Clinical and Experimental Microbiology 2016; 17(4): 243-9.

138. Sharan H, Katare N, Pandey A, Bhatambare GS, Bajpai T. Emergence of hospital acquired carbapenem resistant non fermenters in teaching institute. Journal of Clinical and Diagnostic Research 2016; 10(12): DC20-DC3.

139. Sharan H, Mishra AP, Mishra R. Predictors of Surgical Site Infections in Rural Kanpur, India. Journal of Pure and Applied Microbiology 2013; 7(3): 2309-14.

140. Sharma NK, Garg R, Baliga S, Gopalkrishna BK. Nosocomial infections and drug susceptibility patterns in methicillin sensitive and methicillin resistant Staphylococcus aureus. Journal of Clinical and Diagnostic Research 2013; 7(10): 2178-80.

141. Shimi A, Touzani S, Elbakouri N, Bechri B, Derkaoui A, Khatouf M. Nosocomial pneumonia in ICU CHU Hassan II of Fez. The Pan African medical journal 2015; 22: 285-.

142. Shrestha LB, Baral R, Khanal B. Comparative study of antimicrobial resistance and biofilm formation among Gram-positive uropathogens isolated from community-acquired urinary tract infections and catheter-associated urinary tract infections. Infection and Drug Resistance 2019; 12: 957-63.

143. Shrestha R, Koju P, Liu X, et al. Health care associated infection and trend of antimicrobial resistance in tertiary care hospital-a study in low income setting. Kathmandu University Medical Journal 2019; 17(68): 329-35.

144. Sohail M, Latif Z. Prevalence and antibiogram of methicillin resistant Staphylococcus aureus isolated from medical device-related infections; A retrospective study in Lahore, Pakistan. Revista da Sociedade Brasileira de Medicina Tropical 2017; 50(5): 680-4.

145. Syed U, Shakirullah, Ullah A, et al. Prevalence and antimicrobial susceptibility pattern of ESBL producing gram negative rods causing nosocomial infection. International Journal of Research in Pharmaceutical Sciences 2013; 4(2): 171-6.

146. Taha AE, Badr MF, El-Morsy FE, Hammad E. Prevalence and Antimicrobial Susceptibility of Methicillin-Resistant Staphylococcus aureus in an Egyptian University Hospital. Journal of Pure and Applied Microbiology 2019; 13(4): 2111-22.

147. Talaat M, El-Shokry M, El-Kholy J, et al. National surveillance of health care-associated infections in Egypt: Developing a sustainable program in a resource-limited country. American Journal of Infection Control 2016; 44(11): 1296-301.

148. Tambuwal SH, Iliyasu G, Muhammad Dayyab F, et al. Clinical and Microbiologic Profile of Patients with Staphylococcus aureus Infection. Infectious Diseases in Clinical Practice 2020; 28(2): 78-83.

149. Tolera M, Abate D, Dheresa M, Marami D. Bacterial Nosocomial Infections and Antimicrobial Susceptibility Pattern among Patients Admitted at Hiwot Fana Specialized University Hospital, Eastern Ethiopia. Advances in medicine 2018; 2018: 2127814-.

150. Tran GM, Ho-Le TP, Ha DT, et al. Patterns of antimicrobial resistance in intensive care unit patients: a study in Vietnam. Bmc Infectious Diseases 2017; 17.

151. Trifi A, Abdellatif S, Oueslati M, et al. Nosocomial infections: current situation in a resuscitation-unit. La Tunisie medicale 2017; 95(3): 179-84.

152. Umer Ul H, Haq A, Waseem R, Waseem W, Nadia S. Microbiological organisms and their antimicrobial sensitivity causing ventilator associated pneumonia [VAP]. Vol. 68, 2018:191-7.

153. van der Meeren BT, Millard PS, Scacchetti M, et al. Emergence of methicillin resistance and Panton-Valentine leukocidin positivity in hospital- and community-acquired Staphylococcus aureus infections in Beira, Mozambique. Tropical Medicine & International Health 2014; 19(2): 169-76.

154. Vasanthi R, Jeya M, Karthick. Inducible clindamycin resistance among community and hospital acquired isolates of staphylococcus species. International Journal of Pharma and Bio Sciences 2012; 3(3): B372-B80.

155. Vien Le M, Nguyen Thi Khanh N, Voong Vinh P, et al. In vitro activity of colistin in antimicrobial combination against carbapenem-resistant Acinetobacter baumannii isolated from patients with ventilator-associated pneumonia in Vietnam. Journal of Medical Microbiology 2015; 64: 1162-9.

156. Vijaya, Saldanha DRM, Shenoy S. Clinico-microbiological study of infections in the intensive care unit and study of antimicrobial resistance in bacterial isolates. 2014.

157. Vijayanarayana K, Rau NR, Naik NA, et al. An appraisal of sensitivity and resistance pattern of organisms isolated from hospital acquired pneumonia patients. Research Journal of Pharmaceutical, Biological and Chemical Sciences 2014; 5(4): 384-98.

158. Vijaykumar S, Nayak NP, Shettigar S, Rai Y. BACTERIOLOGICAL CULTURE OF ENDOTRACHEAL ASPIRATES: A SIMPLE DIAGNOSTIC TOOL AND A GUIDE FOR EMPIRIC ANTIBIOTIC THERAPY IN SUSPECTED CASES OF VENTILATOR-ASSOCIATED PNEUMONIA. Journal of Evolution of Medical and Dental Sciences-Jemds 2016; 5(74): 5445-50.

159. Vipin C, Mujeeburahiman M, Arun AB, Ashwini P, Mangesh SV, Rekha PD. Adaptation and diversification in virulence factors among urinary catheter-associated Pseudomonas aeruginosa isolates. Journal of Applied Microbiology 2019; 126(2): 641-50.

160. Vu Dinh P, Nadjm B, Nguyen Hoang Anh D, et al. Ventilator-associated respiratory infection in a resource-restricted setting: impact and etiology. Journal of Intensive Care 2017; 5.

161. Vu Dinh P, Wertheim HFL, Larsson M, et al. Burden of Hospital Acquired Infections and Antimicrobial Use in Vietnamese Adult Intensive Care Units. Plos One 2016; 11(1).

162. Vu Quoc D, Hieu Ngoc V, Hung Nguyen T, et al. Bacterial bloodstream infections in a tertiary infectious diseases hospital in Northern Vietnam: aetiology, drug resistance, and treatment outcome. Bmc Infectious Diseases 2017; 17.

163. Yazigi H, Khamees A, Wakil H. Acquired urinary tract infection in the public hospital. Research Journal of Pharmacy and Technology 2019; 12(3): 1255-8.
