# Supplementary figures and images for: Antibiotic resistance in hospital-acquired ESKAPE-E infections in low- and lower-middle-income countries: a systematic review and meta-analysis
Source: Emerg Microbes Infect. 2022 Feb 4;11(1):443–51. doi: 10.1080/22221751.2022.2030196 (PMC8820817; doi:10.1080/22221751.2022.2030196)

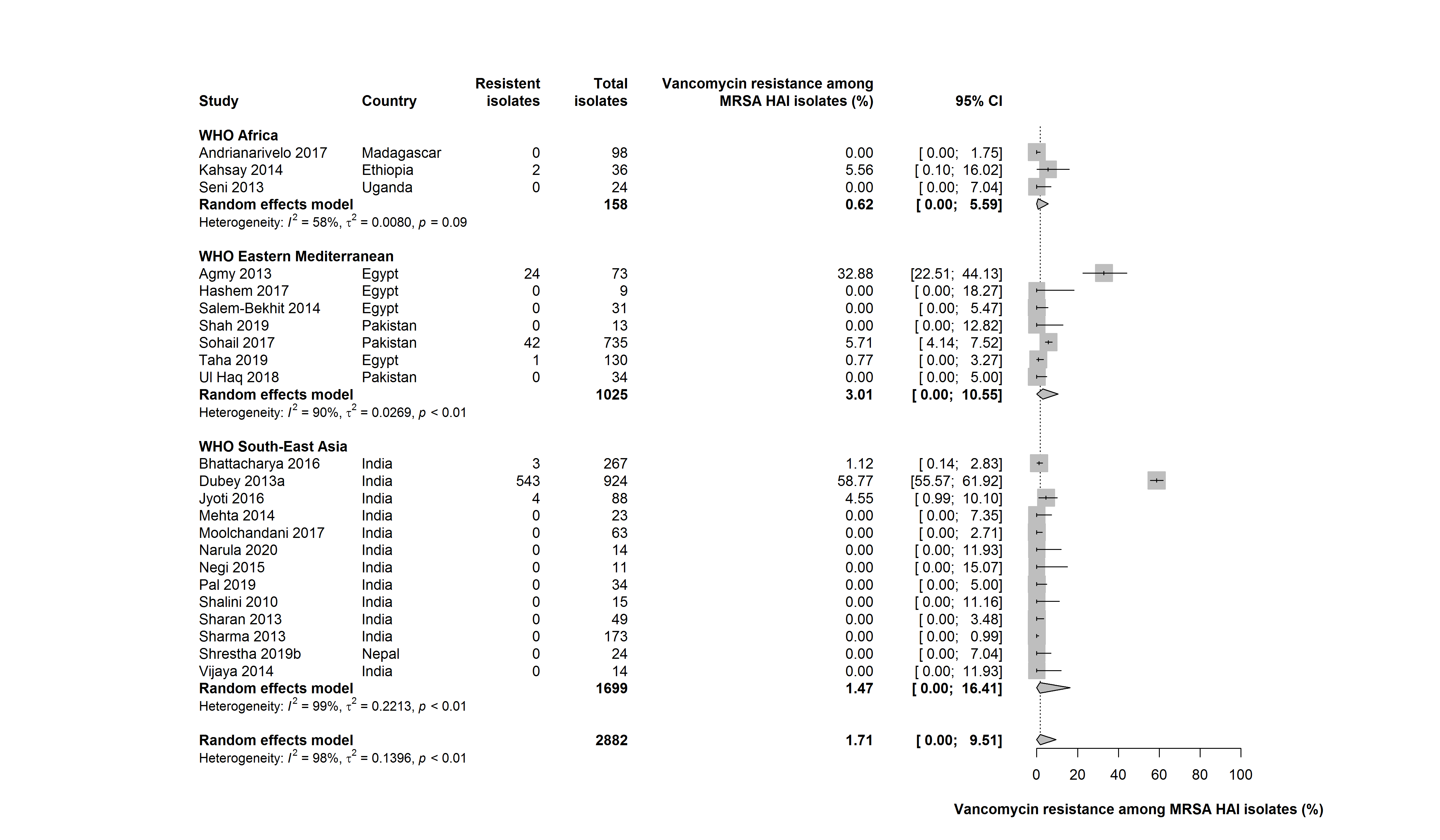

Supplement: Supplemental Material [file TEMI_A_2030196_SM3719.zip › Suppl files/sFigure10-VR-MRSA400.tiff]

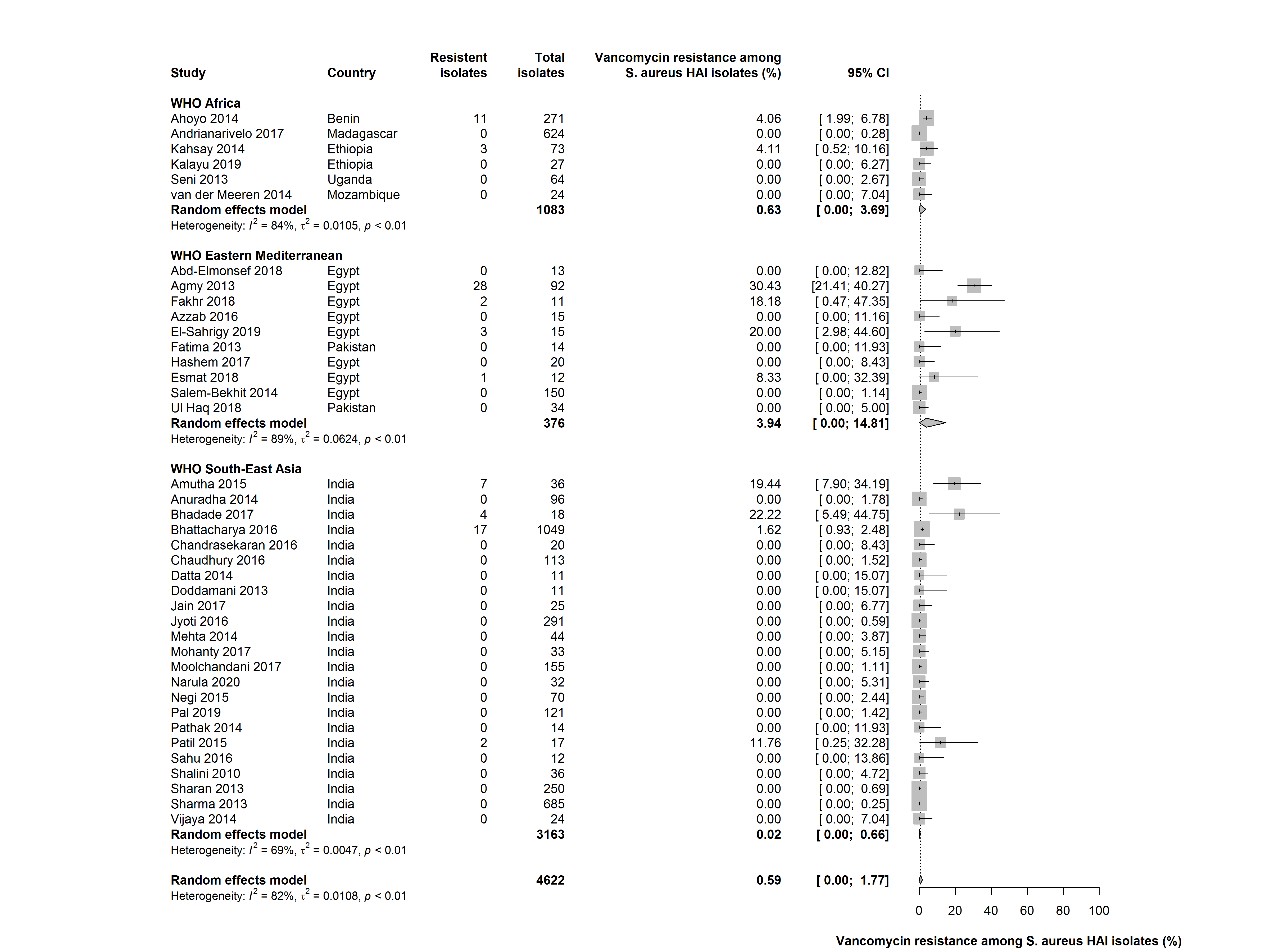

Supplement: Supplemental Material [file TEMI_A_2030196_SM3719.zip › Suppl files/sFigure11-VR-SA400.tiff]

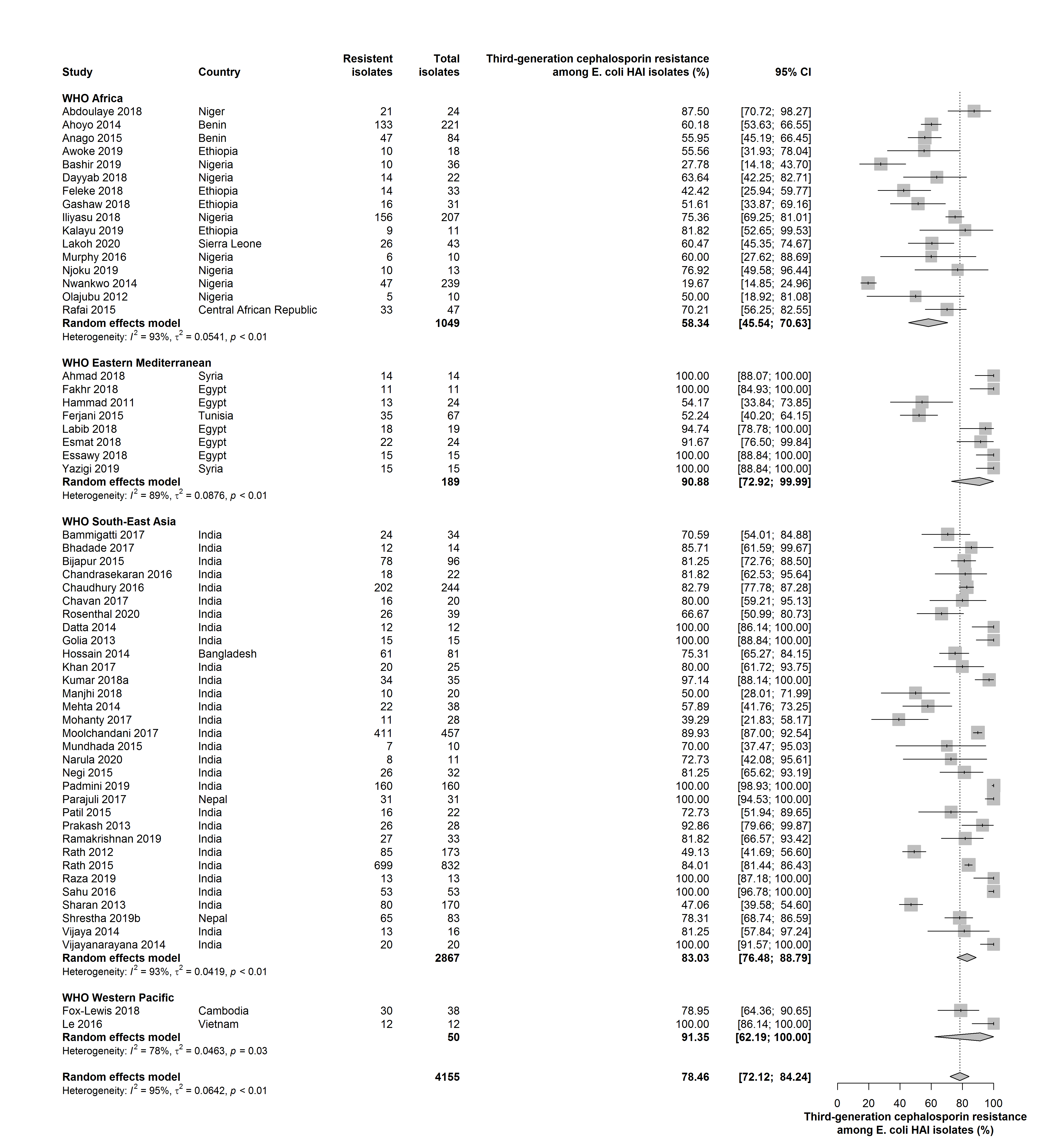

Supplement: Supplemental Material [file TEMI_A_2030196_SM3719.zip › Suppl files/sFigure1-Ceph-EC400.tiff]

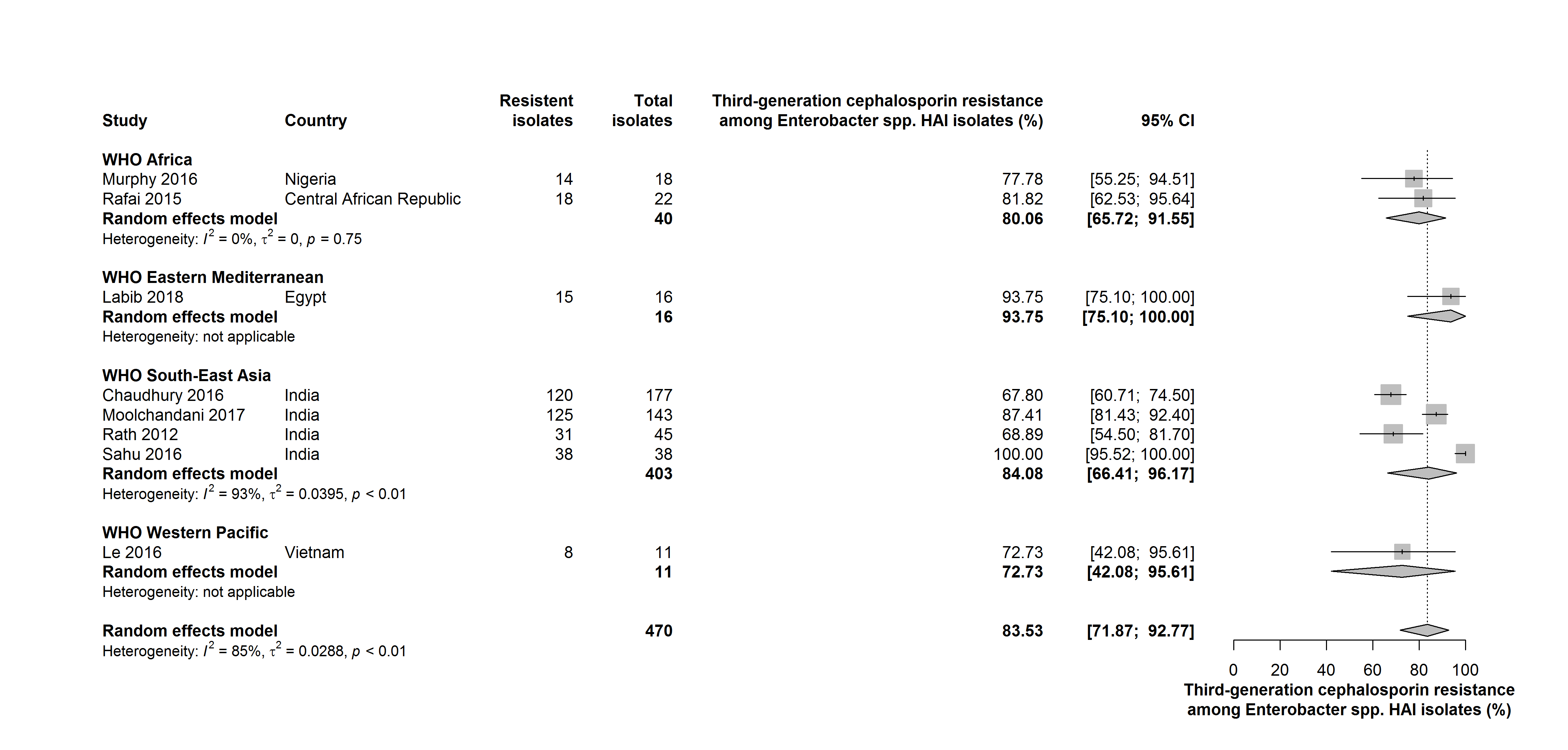

Supplement: Supplemental Material [file TEMI_A_2030196_SM3719.zip › Suppl files/sFigure2-Ceph-Ent400.tiff]

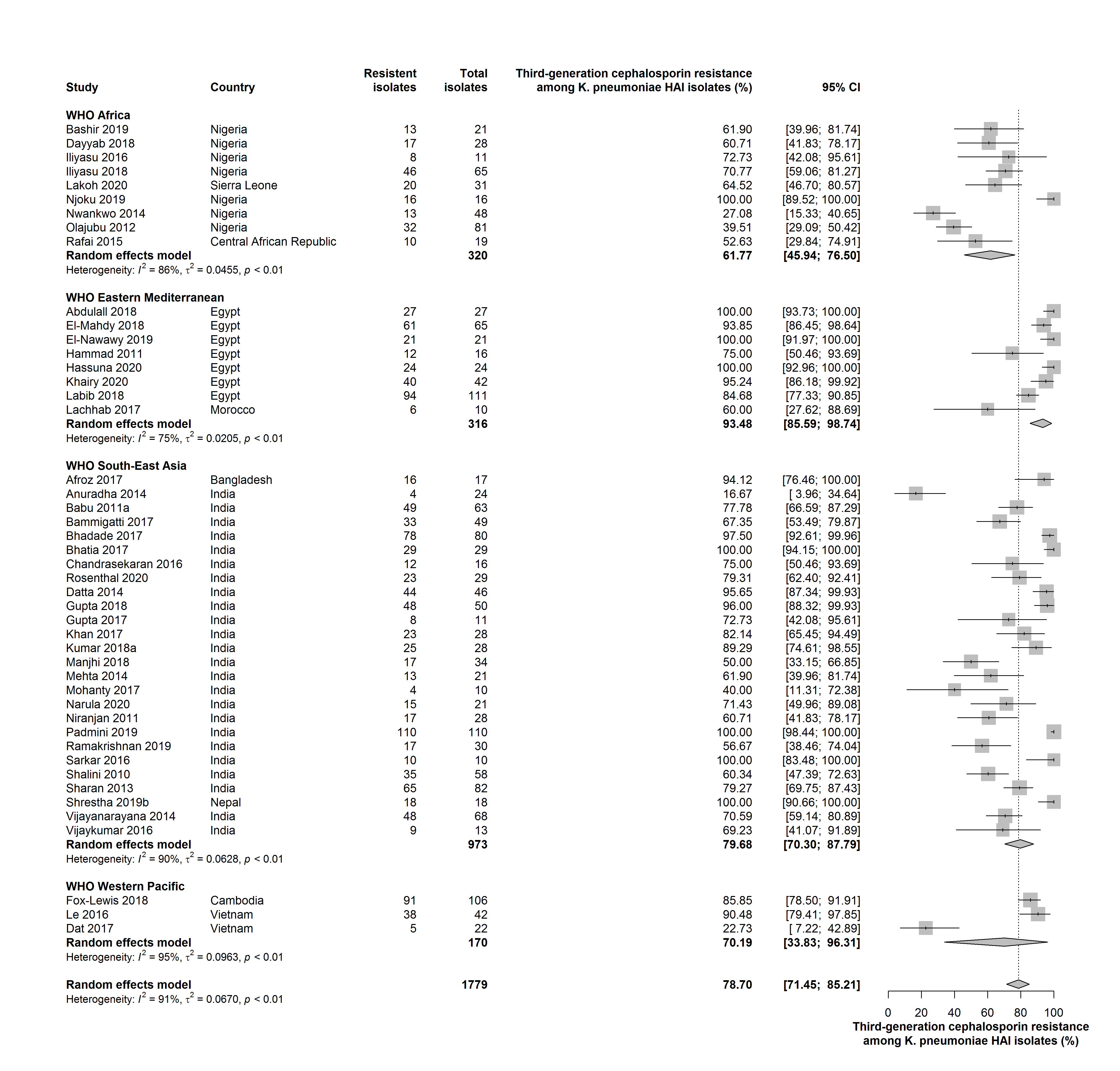

Supplement: Supplemental Material [file TEMI_A_2030196_SM3719.zip › Suppl files/sFigure3-Ceph-KP400.tiff]

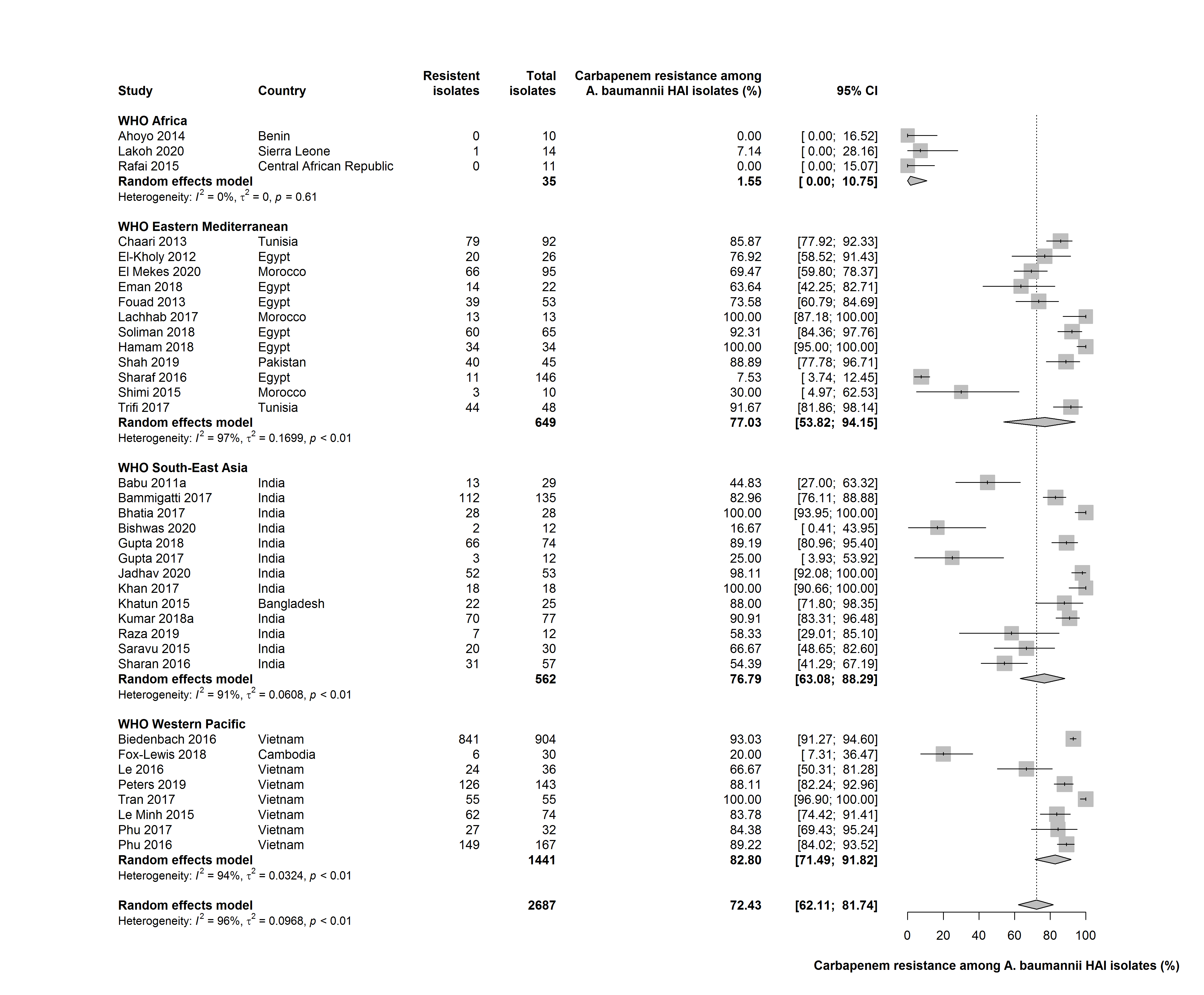

Supplement: Supplemental Material [file TEMI_A_2030196_SM3719.zip › Suppl files/sFigure4-CRABC400.tiff]

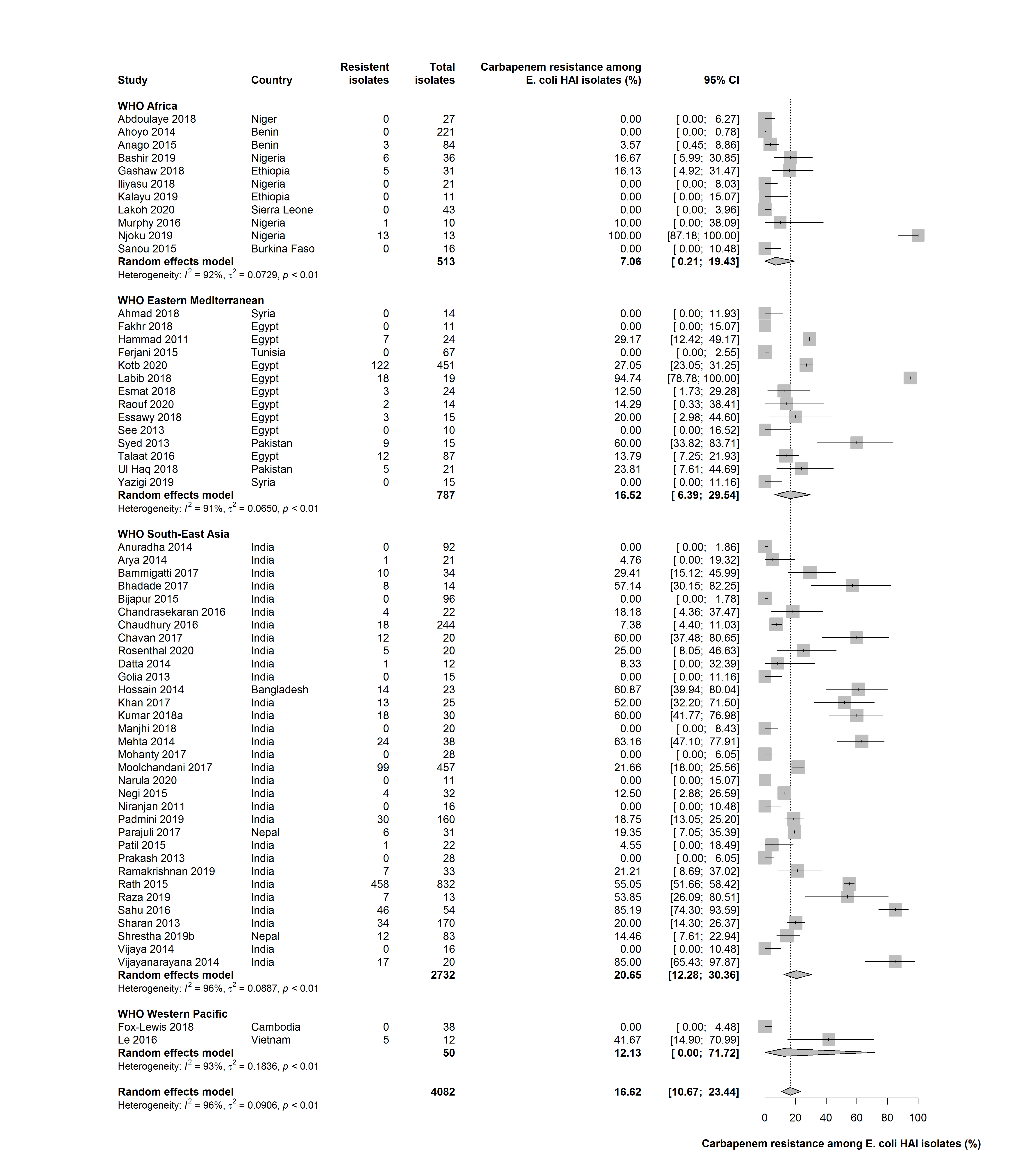

Supplement: Supplemental Material [file TEMI_A_2030196_SM3719.zip › Suppl files/sFigure5-CREC400.tiff]

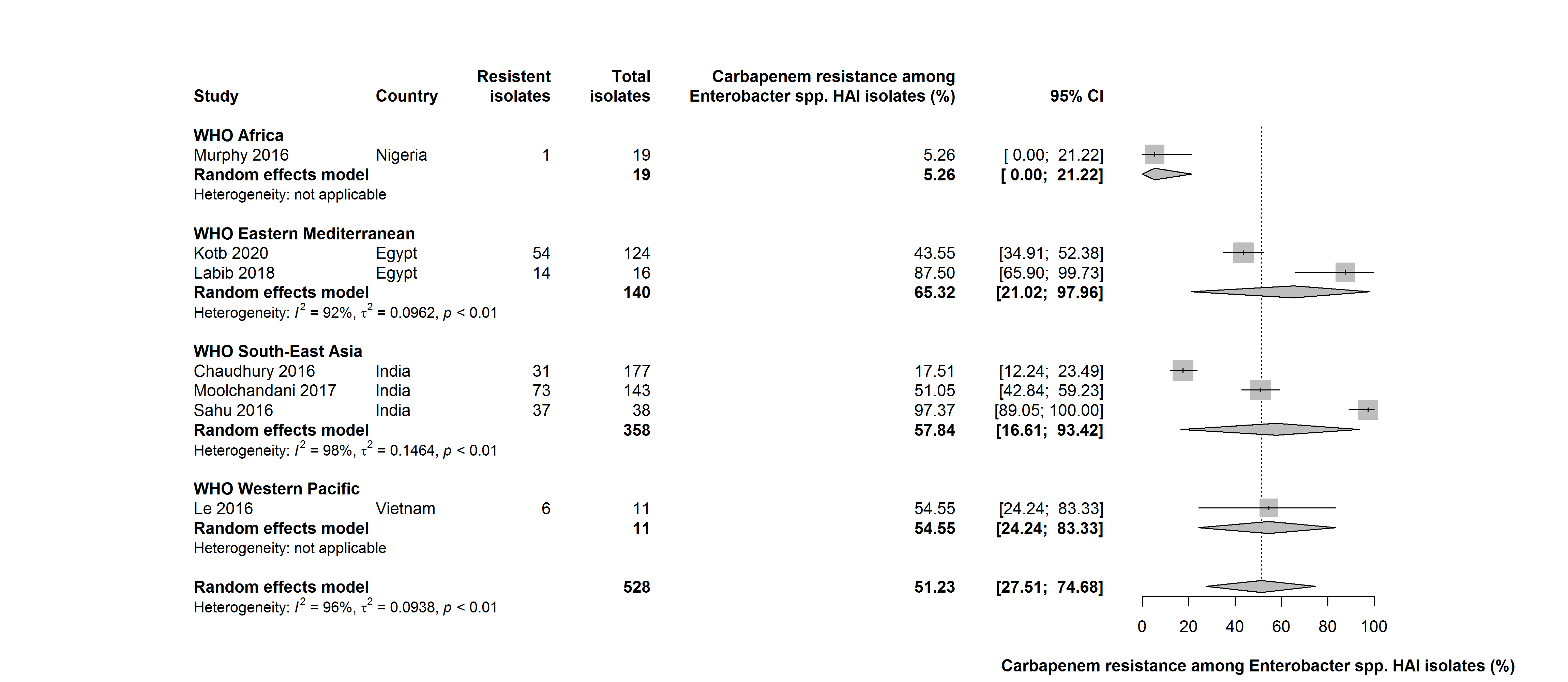

Supplement: Supplemental Material [file TEMI_A_2030196_SM3719.zip › Suppl files/sFigure6-CREnt400.tiff]

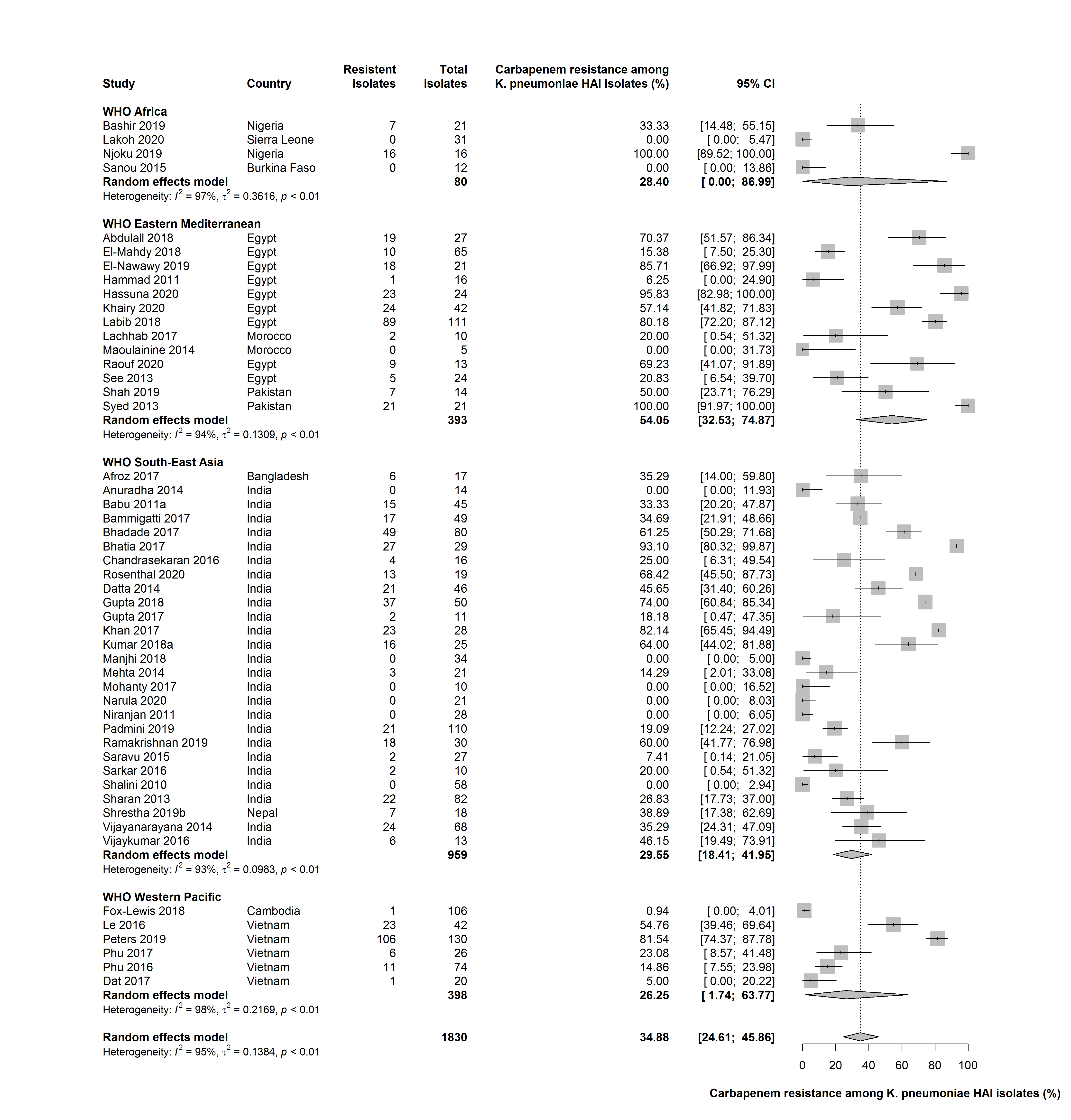

Supplement: Supplemental Material [file TEMI_A_2030196_SM3719.zip › Suppl files/sFigure7-CRKP400.tiff]

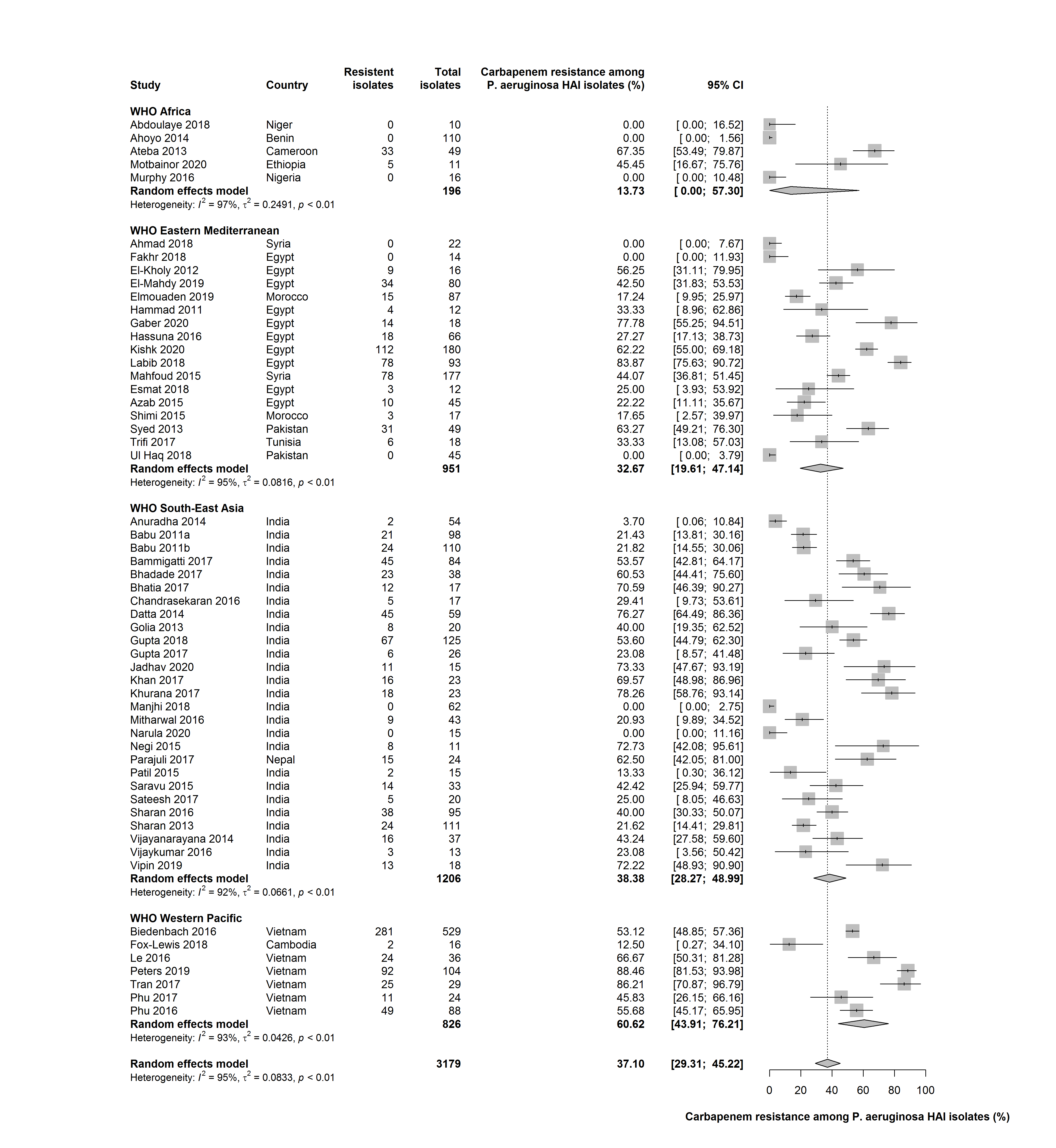

Supplement: Supplemental Material [file TEMI_A_2030196_SM3719.zip › Suppl files/sFigure8-CRPA400.tiff]

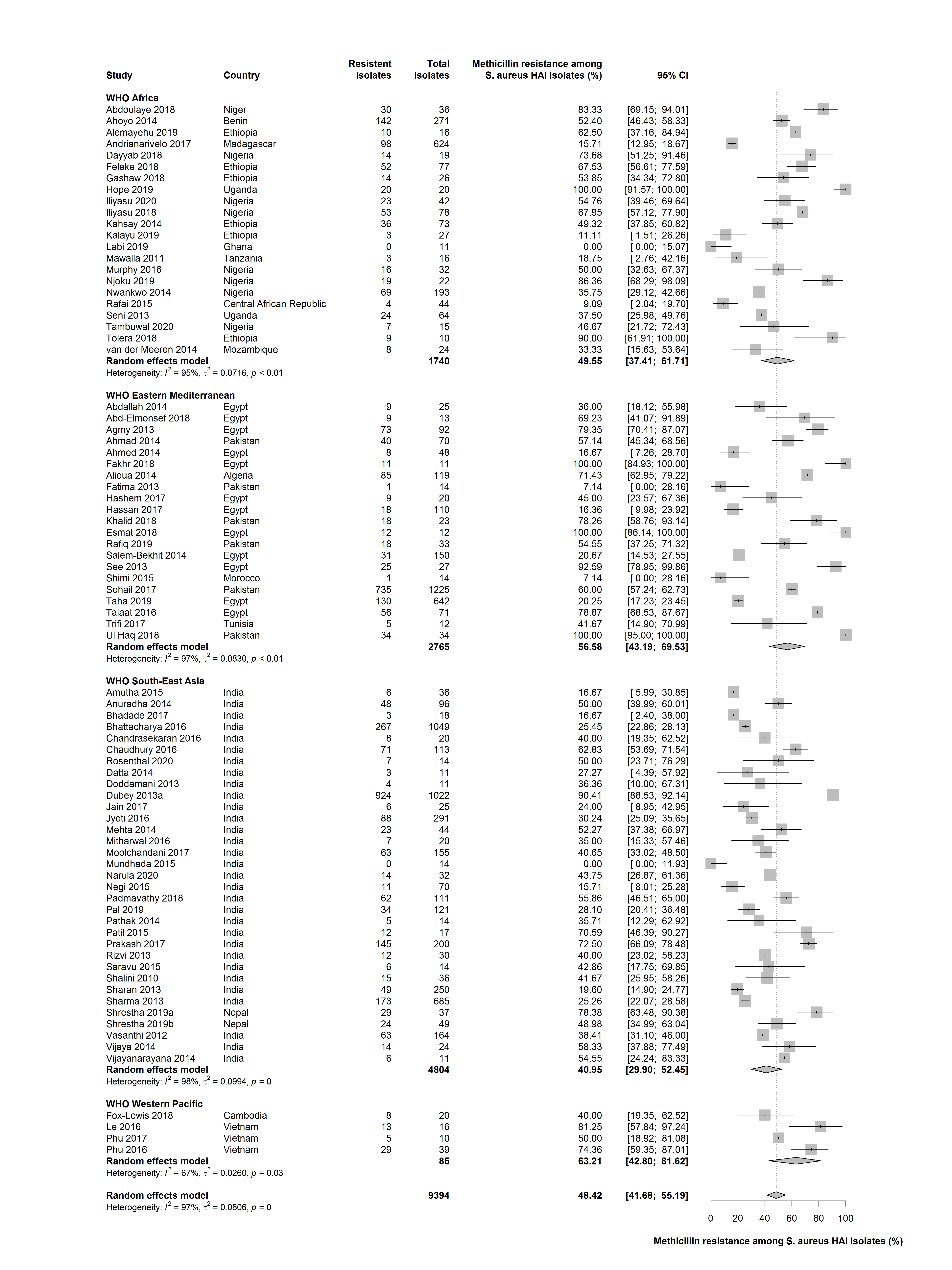

Supplement: Supplemental Material [file TEMI_A_2030196_SM3719.zip › Suppl files/sFigure9-MRSA400.tiff]
